# Stimulated Emission from 2D CdSe/CdS Nanoplatelets Integrated in a Liquid-Core Fiber

**DOI:** 10.1021/acs.nanolett.5c05747

**Published:** 2026-03-06

**Authors:** Veronika Adolfs, Dominik A. Rudolph, Simon Spelthann, Artsiom Antanovich, Dan H. Chau, Mario Chemnitz, Markus A. Schmidt, Jannika Lauth, Michael Steinke

**Affiliations:** § Institute of Quantum Optics, 26555 Leibniz University Hannover, Welfengarten 1, D-30167 Hannover, Germany; ⊥ Cluster of Excellence PhoenixD (Photonics, Optics, and Engineering − Innovation Across Disciplines), Welfengarten 1A, D-30167 Hannover, Germany; ∥ Institute of Physical Chemistry and Electrochemistry, Leibniz University Hannover, Callinstraße 3A, D-30167 Hannover, Germany; # Laboratory of Nano and Quantum Engineering (LNQE), Leibniz University Hannover, Schneiderberg 39, D-30167 Hannover, Germany; ¶ Leibniz Institute of Photonic Technology, Albert-Einstein-Straße 9, D-07745 Jena, Germany; $ Institute of Applied Optics and Biophysics, Philosophenweg 7, D-07743 Jena, Germany; ○ Otto Schott Institute of Material Research, Fraunhoferstraße 6, D-07745 Jena, Germany; ∇ Institute of Physical and Theoretical Chemistry, University Tübingen, Auf der Morgenstelle 18, D-72076 Tübingen, Germany; ◆ QUEST-Leibniz-Research School, Leibniz University Hannover, Callinstraße 36, D-30167 Hannover, Germany

**Keywords:** nanoplatelets, colloidal, liquid-core fiber, biexcitons, stimulated emission

## Abstract

Colloidal nanocrystals are unique optical gain materials due to their high intrinsic absorption, excellent quantum yield, and tunable emission. However, integration of colloidal nanocrystal solutions into photonic systems for lasing applications is challenging since high concentration levels are required for optical amplification. Here, we address this challenge by integrating colloidally dispersed core/crown CdSe/CdS 2D nanoplatelets in liquid-core optical fibers as a scalable platform. The platform allowed achievement of sufficient gain for amplified spontaneous emission at a threshold as low as 1.8 kW/cm^2^ under quasi-CW pumping, even at a concentration two orders of magnitude lower than the concentration levels considered to be required for gain with conventional colloidal 0D quantum dots. We show that the low-loss optical waveguiding of the fiber is crucial for efficient stimulated emission, rendering liquid-core fibers as a promising and unique platform to realize lasers based on colloidally dispersed nanocrystals.

## Graphical abstract

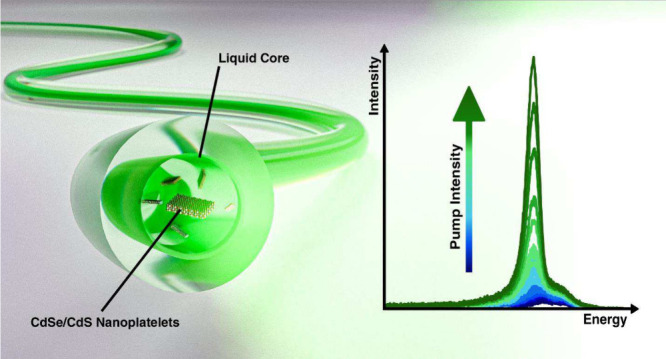

Colloidal nanocrystalline emitters represent a promising optical gain medium and have been studied in various configurations and material compositions.^1–3^ Among these emitters, 2D semiconductor nanoplatelets (NPLs) stand out because they exhibit high intrinsic absorption cross sections,^4,5^ narrow emissions ranging from the ultraviolet to the near-infrared,^6–11^ and low thresholds for amplified spontaneous emission (ASE) and lasing.^12–21^ CdSe NPLs with a CdS crown constitute a comparably simple heterostructure with emission wavelengths tunable via the number of atomic layers.^22–24^ The core/crown design leads to high photoluminescence (PL) quantum yields since nonradiative recombination at edge defect states is efficiently suppressed.^22–25^ Additionally, in comparison to core-only NPLs, superior performance with respect to ASE and low lasing thresholds have been demonstrated with core/crown NPLs.^12^

To date, ASE or lasing with semiconductor nanomaterials has mainly been realized in solid thin films, due to ease of preparation and high packing densities typically required for gain.^12–20,26–28^ However, such films can be prone to various loss channels such as scattering or Förster resonance energy transfer and subsequent charge carrier trapping.^29^ These challenges can be alleviated by using dispersed nanocrystals in a liquid environment. Corresponding optofluidic setups also offer further advantages, including a relatively straightforward exchange of the gain medium, to either replenish it to prevent degradation^30^ or to change the emission wavelengths.^13^ Despite the advantages, achieving optical gain from colloidal solution is challenging mainly due to the limited nanocrystal solubility. According to an estimation by Park et al.,^31^ when using isotropically confined 0D nanocrystals such as quantum dots, a semiconductor volume fraction of ∼2% is required to overcome inherent nonradiative Auger recombination, even without considering any additional loss factors. Beyond that, the nanomaterial concentration required for gain must also compensate optical losses incurred by the photonic environment. To circumvent these challenges, either specific solvents with high nanocrystal solubility have to be used,^32^ nanomaterials with low nonradiative losses (such as 2D NPLs^33^) have to be considered and/or the optical device itself has to provide a low-loss environment. Consequently, only few studies have been published reporting ASE or lasing in colloidal nanomaterial solutions employing cuvettes or short capillaries.^21,32,34–36^ This demonstrates that, while the maturity of the NPL design and synthesis is quite evolved and well-controlled, scalable low-loss platforms for their optical integration in solution are missing so far.

As an innovative approach to enhance the effective NPL gain even at low concentrations, we propose a scalable platform for the optical integration of colloidal nanocrystals such as NPLs. Particularly, we utilize optical fibers, which efficiently collect the NPL emission and confine it on a small cross-section but are easily scalable in the longitudinal direction due to low-loss waveguiding. Fibers based on fused (vitreous) silica offer excellent technological maturity and chemical, thermal, and mechanical robustness.^37^ Such fibers form the basis of modern data communication^38^ and active fibers doped with trivalent lanthanide ions enable kW-class continuous-wave (CW) or high-energy pulsed laser systems.^39^ However, pronounced multiphonon quenching of fused silica prevents visible lasing from lanthanide-doped fibers^40^ and, in turn, makes nanocrystals a promising wavelength-tunable laser gain medium. While direct doping of colloidal nanocrystals into fused silica cannot be achieved at the extreme temperatures above 2000 °C required for fiber fabrication,^41^ liquid-core fibers (LCFs) overcome their solid counterparts’ disadvantage and open a promising path for the photonic integration of the nanocrystals.^42^ Such LCFs have recently received substantial interest, particularly in nonlinear photonics due to unique properties such as noninstantaneous nonlinear response^43^ or the ability to tune dispersion in real-time through temperature modulation.^44^ Specifically, nanomaterial dispersions can be straightforwardly introduced into LCFs under ambient conditions via capillary action. Recently, Zhang et al. followed this idea by demonstrating gain from quantum dots embedded in an LCF.^45^ However, due to the high competition between radiative emission and nonradiative Auger recombination in 0D quantum dots, a complex ternary-alloyed CdZnSe/ZnSeS/ZnS structure with a precisely engineered core/shell interface and concentrations in the range of hundreds of grams per liter were required to achieve this result. NPLs, on the other hand, offer practical advantages including a low inherent ASE threshold and low Auger recombination rates arising from their 2D structure and the strong confinement.^28,46^

Here, for the first time, we integrate core-crown NPLs in LCFs and demonstrate ASE (synonymously referred to as stimulated emission) under quasi-CW pumping. Using a numerical spectral decomposition, we find that optical gain can only build up for red-shifted biexcitons, an observation we put into context with two common explanations of the gain mechanism in NPLs. We highlight that the low-loss waveguiding of the LCFs is a unique approach to obtain gain, even at comparably low NPL concentrations. The impact of our results is 2-fold:

(1) Our approach opens a promising path for significantly extending the spectral regime of fiber lasers toward the visible wavelength range.

(2) LCFs offer a scalable, chemically inert, and environmentally robust platform for photonic integration of NPLs and further colloidal nanocrystalline emitters.

Within this work, we used 4.5 monolayer thick CdSe/CdS core/crown NPLs at a low concentration of ∼1.56 μmol/L (∼1.02 g/L; volume fraction of ∼0.015%) prepared according to published procedures (see Section S2).^6,22,23,47^ The NPLs have a rectangular shape with an average lateral size of 27 × 9 nm^2^ (Figures 1a and S1a), starting from an average CdSe core size of 14 × 4 nm^2^ (Figure S1b). Figure 1b shows the absorption and the PL of the NPLs as measured in a cuvette. The latter is centered at 2.41 eV (∼515 nm) with a full width at half-maximum (FWHM) of 39 meV and features a quantum yield of 85%. The NPLs were dissolved in tetrachloroethylene (TCE), which was used due to its high boiling point (121 °C), high transparency, and high refractive index of 1.51 (at 530 nm),^48^ which enables waveguiding within LCFs via total internal reflection. Figure 1c shows the evolution of the recorded NPL emission spectra from an LCF with a 26 μm core. Since there is no longitudinal cavity around the LCF, the recorded NPL emission is the result of a single pass propagation though the LCF. In direct comparison with the excitonic (monocomponent) PL of the NPLs, we find a pronounced additional red-shifted (30–45 meV) emission. It indicates the formation of biexcitons and ASE as it has been observed for similar NPL systems.^12,49^

**Figure 1:**
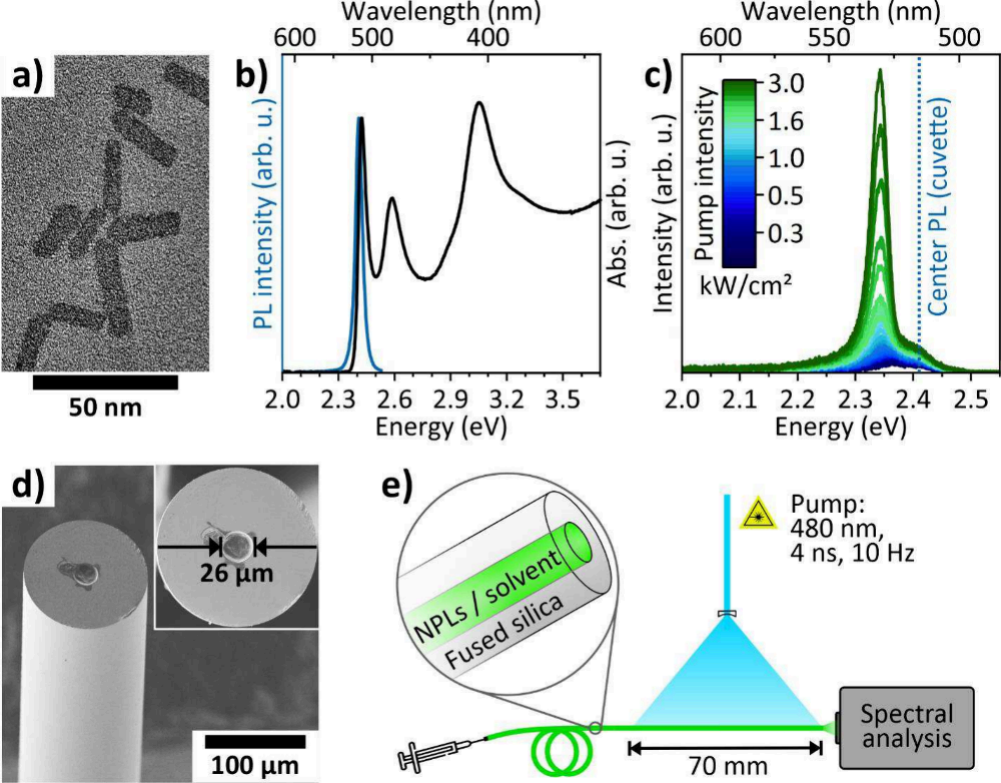
(a) TEM image of the colloidal 2D CdSe/CdS core-crown NPLs. (b) Absorbance (black) and PL (blue) of the NPLs in TCE. (c) Emission spectra obtained from an LCF filled with a colloidal solution of CdSe/CdS NPLs under an increasing intensity of the quasi-CW pump laser. (d) SEM image of the LCF used in the experiments. (e) Scheme of the setup used to laterally pump the fiber by a quasi-CW laser system.

A SEM picture of the LCF and a schematic sketch of the setup used to obtain our results is presented in Figure 1d,e (also see Section S4). We transversally pumped a 7 cm long section of the LCF by an optical parametric oscillator tuned to 480 nm. The pump laser provides 4 ns pulses with an energy density of up to 21 μJ/cm^2^, which corresponds to a maximum (peak) intensity of 5.4 kW/cm^2^ (note that the pump pulses are 5 orders of magnitude longer than fs-lasers that are commonly used to pump the short-lived excited NPL states).^12,13,15,27^ Since the expected biexciton (gain) lifetime lies in the order of a few hundred picoseconds^50–52^ —about 10 times shorter than our pulse duration—our experiments are operated in the quasi-CW regime.

To analyze the measured spectra and confirm the onset of ASE, we numerically decomposed the spectra by a model consisting of a Lorentz profile for the excitonic (X) and a pseudo-Voigt profile for the biexcitonic (XX) component, as it has been introduced by Grim et al.^53^ Figure 2a shows a representative result of our fitting procedure. Although the model leads to a somewhat underfitted low-energy tail, no additional features were added since its simplicity allows to capture and explain the relevant gain effects, see below. For an increasing pump intensity, the integrated signals (slopes) of the two fitted components and their center energies reveal two operation regimes, see Figure 2b. The first regime corresponds to pump intensities below 1.8 kW/cm^2^, where the slopes increase sublinearly (exponent of 0.6 for the biexcitonic slope) corresponding to spontaneous excitonic and biexcitonic emission. The second regime corresponds to pump intensities above 1.8 kW/cm^2^, where the biexcitonic slope is significantly steeper (superlinear, exponent of 1.6) than before. Such a pronounced increase of the slope efficiency is a clear indicator of ASE.^12,13,21,36^ Note that the scaling of the slope (sublinear below and superlinear above the threshold) is similar to the CW-pumped system reported by Grim et al.^53^ (see Section S6.5). In terms of temporal stability, we typically observed a decreasing emission after a few minutes. This is not yet quite comparable to CW-pumped setups reported before^53^ but our system offers plenty of room for future optimization. The threshold of 1.8 kW/cm^2^ (as computed by linear fits to the experimental data, see Section S6.5) corresponds to an energy density of 7.2 μJ/cm^2^, which is significantly lower than the previous results of dispersed nanocrystals (1 mJ/cm^2^ for quantum dots in an LCF from Zhang et al.,^45^ 30 μJ/cm^2^ for red-emitting NPLs and 44 μJ/cm^2^ for green-emitting NPLs in short capillary tubes from Delikanli et al.^21^). Note that this comparison is rather qualitative as those previous results were achieved with fs-pumping, whereas we use quasi-CW pumping. The ASE threshold can also be identified in the evolution of the excitonic and biexcitonic center energies and the spectral coherence, see Figure 2b. At the lowest pump power level, the excitonic and biexcitonic center energies are separated by ∼46 meV. Toward the threshold, the biexcitonic center energy shifts further into the red until it remains constant at 2.34 eV (∼530 nm) above the threshold, with a separation of ∼65 meV with respect to the excitonic emission. This means, the ASE emerges on the red tail of the spontaneous biexcitonic emission (see further fitting results in the Supporting Information, SI). In parallel, the spectral coherence of the biexcitonic component also increases as indicated by the narrowing of the FWHM, which is yet another indication of ASE (note that the excitonic component does not show such narrowing). Compared to the FWHM of the measured PL (cuvette), the mean fitted FWHM of the excitonic component shows a slightly lower value of 26 meV, which might be due to reabsorption along the fiber (of the high-energy tail of the PL) and the nonunity quantum yield.

**Figure 2:**
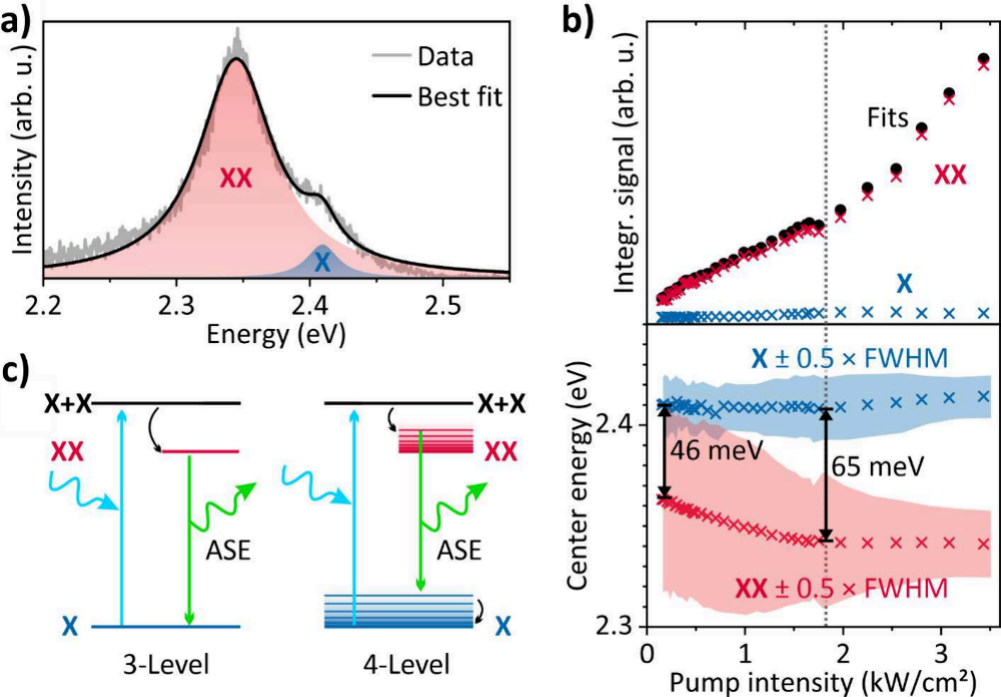
(a) Exemplary decomposition of the recorded LCFs’ emission spectrum at a pump intensity of ∼1.8 kW/cm^2^ into two spectral components, showing an excitonic (X) and biexcitonic (XX) contribution. (b) Evolution of the spectrally integrated emissions (top) and the center energies plus the FWHM of the fitted X and XX components (bottom). The dashed line indicates an ASE threshold of 1.8 kW/cm^2^. (c) Scheme of the relevant NPL states involved in the optical amplification process in a three-level (left) and a four-level (right) system.

The red-shift of the ASE can be understood when considering two common but complementary explanations of how gain in NPLs occurs.^54,55^ From a laser physics perspective, gain originates from either a three-level system or a four-level system, as shown in Figure 2c. In the three-level system, the requirement for gain is an average biexciton density ⟨N_*xx*_⟩ that is larger than the exciton density ⟨N_*x*_⟩. This explanation is directly adapted from the common understanding of the processes in quantum dots, where gain is achieved at an exciton density ⟨N_*x*_⟩ greater than 1.15 and where two excitons are assumed to always form a biexciton.^54^ However, this picture does not allow for an energy shift of the stimulated emission with respect to the spontaneous biexcitonic emission and cannot explain our observation of a red-shifted ASE. Such a red-shift occurs, however, if the 2D mobility (momentum) of the excitons and biexcitons and their corresponding kinetic energy is considered, as has been demonstrated by Geiregat et al.^55^ They argued that (1) biexcitons and excitons in NPLs exist in a thermodynamic equilibrium and (2) their kinetic energy states are occupied following a Boltzmann distribution. This means, thermally occupied biexcitonic states can provide gain at lower energies (i.e., red-shifted) than the spontaneous biexcitonic emission, even at densities ⟨N_*xx*_⟩ less than ⟨N_*x*_⟩, which is in perfect agreement with our observation. The shift of around 19 meV we observed is consistent with the results by Geiregat et al.^55^ An additional discussion on how the analogy to a four-level system can be drawn is provided in Section S5. Furthermore, we show (Section S6.1) that it is impossible that the red-shift of the ASE originates from a spectral filtering or a Purcell effect caused by the density of states of the guided modes in the LCF.

The recorded spectra and the fitted slopes (Figure 2b), however, imply that there are significantly more biexcitons than excitons, i.e., that ⟨N_*x*_⟩ is actually less than ⟨N_*xx*_⟩. This seems to be in contradiction with the explanation provided by Geiregat et al. but can be resolved by the following two arguments. (1) Due to the quasi-CW pumping scheme, we cannot reliably anticipate the expected exciton and biexciton densities. However, additional calculations in the SI demonstrate that ⟨N_*xx*_⟩ is expected to be less than ⟨N_*x*_⟩ even assuming a best-case scenario. This scenario corresponds to an infinite charge carrier lifetime, i.e., charge carriers accumulate during the pump pulse without decay. (2) We provide an additional analysis in Figure S9, indicating high kinetic energies of the biexcitons that correspond to effective temperatures up to 500 K above room temperature. Assuming that excitons will experience similar temperatures, a high number of them are expected to be in momentum-forbidden dark states, i.e., the quantum yield of their emission will be significantly reduced. In contrast, such momentum-forbidden dark states do not exist for biexcitons. The combination of these two arguments explains the seemingly high relative number of biexcitons implied by the slopes of the fitted excitonic and biexcitonic emissions (Figure 2b). The nonexistence of momentum-forbidden biexcitonic dark states also provides a possible explanation for the large FWHM of the fitted biexcitonic component up to 90 meV, also see Section S5. However, other effects cannot be excluded at this point and further investigations on the broad biexcitonic emission are required.

Since the photonic environment provided by the LCF is crucial for the generation of stimulated emissions, we performed further investigations of the LCF parameters. First, we conducted experiments with NPLs dispersed in hexane to suppress the waveguiding of the fiber since the refractive index of hexane is 1.38 at 530 nm,^56^ which is below that of fused silica (1.46 ^57^). For brevity, we will refer to the LCF filled with hexane as a nonwaveguiding fiber to distinguish it from the waveguiding fibers filled with NPLs dispersed in TCE. The slope of the nonwaveguiding fiber, shown in Figure 3a, obtained by integrating the recorded spectra, coincides well with the corresponding slope of the waveguiding fiber up to its ASE threshold (1.8 kW/cm^2^). This is expected because even for the waveguiding fiber, spontaneous emission originates primarily from the fiber tip because any such emission upstream of the fiber is effectively reabsorbed along its length. Only at the ASE threshold, the emission in the waveguiding fiber is sufficiently red-shifted to reach stimulated emission. However, the slope obtained from the nonwaveguiding fiber lacks any indication of stimulated emission. It even rolls off at the highest pump intensities, which we attribute to an increasing number of excitons in momentum-forbidden dark states (see discussion above). We numerically decomposed the spectra obtained from the nonwaveguiding fiber into two components (X and XX) and show their corresponding center energies and the FWHM in Figure 3b (also see Section S6.4). The lack of a threshold is also supported by the comparably low excitonic to biexcitonic energy separation of 30 meV (lowest pump intensity) and 45 meV (highest pump intensity), which does not increase as significantly as for the waveguiding fiber (see Figure 2b for comparison). In addition and also in contrast to the waveguiding fiber, no narrowing of the FWHM (increase of the spectral coherence) can be observed.

**Figure 3:**
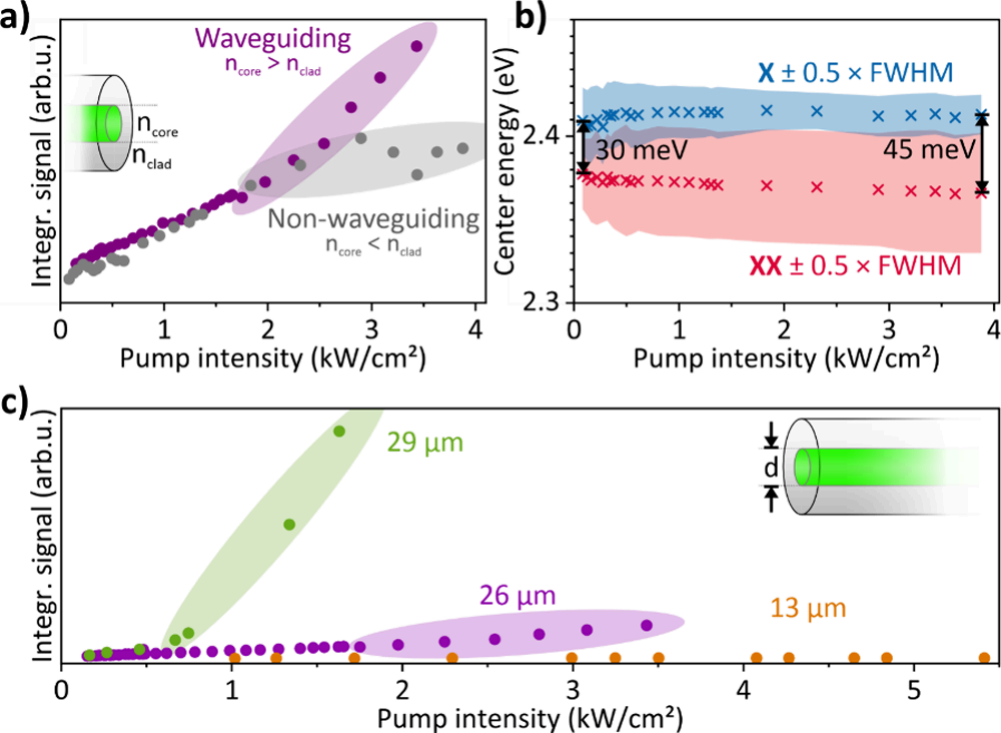
(a) Integral emission intensity in LCFs filled with CdSe/CdS NPLs dispersed in hexane (no waveguiding) and TCE (waveguiding). (b) Evolution of the center energies of the fitted excitonic and biexcitonic components for the hexane-filled fiber. (c) Slopes obtained from the LCFs with different inner diameters of 13 μm (orange), 26 μm (purple), and 29 μm (green), demonstrating the unique scaling properties provided by LCFs.

Even with a waveguiding fiber, ASE only occurs if the effective (single pass) gain provided by the LCF is sufficiently high to overcome the losses of the system—a requirement that depends on the parameters of the setup. As an example. we repeated our initial experiment using a waveguiding, i.e., TCE-filled, LCF with a smaller core diameter of 13 μm (Figure 3c). For this core size, we did not observe any indication of ASE even at the highest pump intensities, certainly because the absolute number of NPLs and their corresponding effective gain was too small to overcome the losses. Indeed, the three main parameters to tune the threshold and scale the slope efficiency are the LCF core diameter, the fiber loss, and the NPL concentration. To further showcase the influence of the fiber diameter, we repeated our experiment with the same NPL concentration of 1.56 μmol/L (*c*_1_) but with a waveguiding LCF with 29 μm core diameter and compare the result with the former experiment (using the 26 μm core LCF) in Figure 3c. Strikingly, the ASE threshold decreases significantly by around 60% to a pump intensity of 0.7 kW/cm^2^. In parallel, the slope efficiency increases by a factor of around 18. This drastic improvement in the performance can be attributed to an 11.5% increase of the LCF core cross-section, resulting in a 25% increase of the absolute number of NPLs, i.e., effective gain per unit fiber length. The relevance of this parameter is also demonstrated by additional experiments that have been conducted with a constant fiber core diameter of 26 μm and varying NPL concentration (see Section S7). When the concentration is doubled to 3.12 μmol/L (2*c*_1_), the threshold decreases to approximately 35% of the threshold obtained with the concentration c_1_. This result is consistent to the experiment with the increased fiber core. In contrast, when the concentration is halved to 0.78 μmol/L (0.5*c*_1_), no ASE can be observed as it was the case for the reduced fiber core. Furthermore, we determined the background losses of the LCFs with the 26 and 29 μm core (see Section S3.4). Due to differences in the fiber fabrication process, the 29 μm LCF features a waveguide loss of 0.005 dB/cm, which is 1 order of magnitude lower than the loss of the 26 μm LCF (0.05 dB/cm). Indeed, besides the larger cross section, the lower loss also positively influences the threshold and slope efficiency. The numerical decomposition analysis for the 29 μm LCF shows (Figure S10) that the ASE threshold is reached once the biexcitonic center energy is shifted to ∼2.34 eV, which is the same result as for the 26 μm LCF. Likewise, the shifting center energy is accompanied by a sharp increase of spectral coherence, as indicated by the FWHM (Figure S12). Noteworthy, the ASE is separated by 59 meV (65 meV for the 26 μm LCF) from the excitonic emission at the threshold. These results indicate a similar role of the excitons and biexcitons in the gain process, independent of the LCF parameters. However, future investigations will be employed to reveal the correlations of the gain process with the LCF parameters.

In conclusion, we demonstrate a well-implemented, robust, and scalable platform for the photonic integration of nanocrystalline emitters such as 2D NPLs in solution. NPLs are a unique and promising gain material with great potential for optofluidic applications, which to date remain largely unrealized due to performance limitations arising from the inherent low emitter concentration. In this work, we overcome this obstacle by integrating CdSe/CdS core/crown NPLs in waveguiding LCFs. Even at a comparatively low concentration of 1.56 μmol/L and under quasi-CW pumping, we observe ASE above a low threshold of 1.8 kW/cm^2^. We attribute this result to the unique properties of 2D NPLs and their implementation in LCFs as a high-quality photonic platform. To further contextualize our findings, we perform numerical decompositions of the recorded emission spectra, which reveal the role of the excitons and biexcitons. Our analysis aligns well with the NPL gain model by Geiregat et al.^55^ Furthermore, we have investigated the impact of the photonic environment on the ASE enabled by altering the LCF core size. We show that thresholds and slope efficiencies can be positively affected by increasing the fiber diameter and decreasing the fiber loss. Likewise, the NPL concentration also influences the threshold and the slope efficiency. The parameters of the LCFs (length, loss, core diameter) can readily be scaled and adjusted further toward CW pumping with affordable diode lasers—a circumstance that was part of the significant success of lanthanide-doped fiber lasers. LCFs can also be fusion spliced and combined with standard fiber technology^42,58^ and adapted to be operated with various nanoemitter classes. Thus, LCFs will certainly be an impactful contribution to the challenge of bringing colloidal nanomaterials into lasing applications. In addition, NPL-filled LCFs constitute a promising path toward highly efficient and wavelength-tunable fiber lasers in the visible spectral regime.

## Supplementary Material



## Notes

Additional information, including the synthesis of CdSe/CdS core/crown NPLs, additional TEM images used to determine NPL dimensions and size histograms of CdSe core-only and CdSe/CdS core/crown NPLs, PL spectra of CdSe/CdS core/crown NPLs in TCE and hexane, absorption spectra of CdSe core-only and CdSe/CdS core/crown NPLs, calculation of the exciton binding energy for CdSe/CdS core/crown NPLs, calculation of the CdSe/CdS core/crown NPLs concentration, fabrication of fused silica fibers, characterization of fibers by SEM, discussion of numerical aperture and V-number for the used LCFs, characterization of fiber loss parameters, details of the optical pump setup, additional discussion on the NPL gain in a 3-level and 4-level system, emission spectra from CdSe/CdS core/crown NPLs from all LCFs (13, 26, and 29 μm) in their respective solvents, discussion of longitudinal and transversal modes as a possible explanation for the red-shifted emission, discussion and characterization of the ratio between excitons and biexcitons of CdSe/CdS core/crown NPLs in LCFs, additional characterization of CdSe/CdS core/crown NPLs in 29 μm TCE-filled and 26 μm hexane-filled LCFs, fitted linear and nonlinear slopes used to determine ASE thresholds, and additional experiments of LCFs with varying NPL concentrations (PDF)

## ‡

Simply Complex Lab, Ruhr-University Bochum, Universitätsstraße 150, D-44801 Bochum, Germany

## Abbreviations

ASE: amplified spontaneous emission
CW: continuous wave
FWHM: full width at half-maximum
LCF: liquid-core fiber
NPL: nanoplatelets
PL: photoluminescence
TCE: tetrachloroethylene

## References

[1] Klimov V. I., Mikhailovsky A. A., Xu S., Malko A., Hollingsworth J. A., Leatherdale C. A., Eisler H.-J., Bawendi M. G. (2000). Optical Gain and
Stimulated Emission in Nanocrystal Quantum Dots. Science.

[2] Yu J., Chen R. (2020). Optical Properties and Applications of Two-Dimensional
CdSe Nanoplatelets. InfoMat.

[3] Ahn N., Livache C., Pinchetti V., Klimov V. I. (2023). Colloidal Semiconductor
Nanocrystal Lasers and Laser Diodes. Chem. Rev..

[4] Achtstein A. W., Antanovich A., Prudnikau A., Scott R., Woggon U., Artemyev M. (2015). Linear Absorption in CdSe Nanoplates: Thickness and
Lateral Size Dependency of the Intrinsic Absorption. J. Phys. Chem. C.

[5] Yeltik A., Delikanli S., Olutas M., Kelestemur Y., Guzelturk B., Demir H. V. (2015). Experimental Determination of the
Absorption Cross-Section and Molar Extinction Coefficient of Colloidal
CdSe Nanoplatelets. J. Phys. Chem. C.

[6] Ithurria S., Tessier M. D., Mahler B., Lobo R. P. S. M., Dubertret B., Efros A. L. (2011). Colloidal Nanoplatelets with Two-Dimensional
Electronic Structure. Nat. Mater..

[7] Di
Giacomo A., Rodà C., Khan A. H., Moreels I. (2020). Colloidal
Synthesis of Laterally Confined Blue-Emitting 3.5 Monolayer CdSe Nanoplatelets. Chem. Mater..

[8] Antanovich A., Yang L., Erwin S. C., Martín-García B., Hübner R., Steinbach C., Schwarz D., Gaponik N., Lesnyak V. (2022). CdSe_x_S_1‑x_ Alloyed Nanoplatelets
with Continuously Tunable Blue-Green Emission. Chem. Mater..

[9] Christodoulou S., Climente J. I., Planelles J., Brescia R., Prato M., Martín-García B., Khan A. H., Moreels I. (2018). Chloride-Induced
Thickness Control in CdSe Nanoplatelets. Nano
Lett..

[10] Mitrofanov A., Prudnikau A., Di Stasio F., Weiß N., Hübner R., Dominic A. M., Borchert K. B. L., Lesnyak V., Eychmüller A. (2021). Near-Infrared-Emitting Cd_x_Hg_1‑x_Se-Based Core/Shell Nanoplatelets. Chem. Mater..

[11] Zhang Z., Sun H. (2021). Manipulation of the
Optical Properties of Colloidal 2D CdSe Nanoplatelets. Advanced Photonics Research.

[12] Guzelturk B., Kelestemur Y., Olutas M., Delikanli S., Demir H. V. (2014). Amplified Spontaneous
Emission and Lasing in Colloidal
Nanoplatelets. ACS Nano.

[13] Zhang Q., Zhu Y., Niu P., Lao C., Yao Y., Liu W., Yang Q.-F., Chu S., Gao Y. (2023). Low-Threshold Single-Mode
Microlasers from Green CdSe/CdSeS Core/Alloyed-Crown Nanoplatelets. ACS Photonics.

[14] Olutas M., Guzelturk B., Kelestemur Y., Yeltik A., Delikanli S., Demir H. V. (2015). Lateral Size-Dependent Spontaneous and Stimulated Emission
Properties in Colloidal CdSe Nanoplatelets. ACS Nano.

[15] She C., Fedin I., Dolzhnikov D. S., Dahlberg P. D., Engel G. S., Schaller R. D., Talapin D. V. (2015). Red, Yellow, Green, and Blue Amplified
Spontaneous Emission and Lasing Using Colloidal CdSe Nanoplatelets. ACS Nano.

[16] Kelestemur Y., Dede D., Gungor K., Usanmaz C. F., Erdem O., Demir H. V. (2017). Alloyed Heterostructures
of CdSe_x_S_1‑x_ Nanoplatelets with Highly
Tunable Optical Gain Performance. Chem. Mater..

[17] Kelestemur Y., Shynkarenko Y., Anni M., Yakunin S., De Giorgi M. L., Kovalenko M. V. (2019). Colloidal CdSe Quantum Wells with Graded Shell Composition
for Low-Threshold Amplified Spontaneous Emission and Highly Efficient
Electroluminescence. ACS Nano.

[18] Yang Z., Pelton M., Fedin I., Talapin D. V., Waks E. (2017). A Room Temperature
Continuous-Wave Nanolaser Using Colloidal Quantum Wells. Nat. Commun..

[19] Dede D., Taghipour N., Quliyeva U., Sak M., Kelestemur Y., Gungor K., Demir H. V. (2019). Highly Stable Multicrown
Heterostructures
of Type-II Nanoplatelets for Ultralow Threshold Optical Gain. Chem. Mater..

[20] Altintas Y., Gungor K., Gao Y., Sak M., Quliyeva U., Bappi G., Mutlugun E., Sargent E. H., Demir H. V. (2019). Giant Alloyed
Hot Injection Shells Enable Ultralow Optical Gain Threshold in Colloidal
Quantum Wells. ACS Nano.

[21] Delikanli S., Erdem O., Isik F., Dehghanpour Baruj H., Shabani F., Yagci H. B., Durmusoglu E. G., Demir H. V. (2021). Ultrahigh Green and Red Optical Gain Cross Sections
from Solutions of Colloidal Quantum Well Heterostructures. J. Phys. Chem. Lett..

[22] Tessier M. D., Spinicelli P., Dupont D., Patriarche G., Ithurria S., Dubertret B. (2014). Efficient
Exciton Concentrators Built
from Colloidal Core/Crown CdSe/CdS Semiconductor Nanoplatelets. Nano Lett..

[23] Schlosser A., Graf R. T., Bigall N. C. (2020). CdS Crown Growth on CdSe Nanoplatelets:
Core Shape Matters. Nanoscale Adv..

[24] Prudnikau A., Chuvilin A., Artemyev M. (2013). CdSe-CdS Nanoheteroplatelets with
Efficient Photoexcitation of Central CdSe Region through Epitaxially
Grown CdS Wings. J. Am. Chem. Soc..

[25] Singh S., Tomar R., ten Brinck S., De Roo J., Geiregat P., Martins J. C., Infante I., Hens Z. (2018). Colloidal CdSe Nanoplatelets,
A Model for Surface Chemistry/Optoelectronic Property Relations in
Semiconductor Nanocrystals. J. Am. Chem. Soc..

[26] Guzelturk B., Kelestemur Y., Olutas M., Li Q., Lian T., Demir H. V. (2017). High-Efficiency Optical Gain in Type-II Semiconductor
Nanocrystals of Alloyed Colloidal Quantum Wells. J. Phys. Chem. Lett..

[27] Grim J. Q., Christodoulou S., Di Stasio F., Krahne R., Cingolani R., Manna L., Moreels I. (2014). Continuous-Wave Biexciton Lasing
at Room Temperature Using Solution-Processed Quantum Wells. Nat. Nanotechnol..

[28] Diroll B. T., Talapin D. V., Schaller R. D. (2017). Violet-to-Blue
Gain and Lasing from
Colloidal CdS Nanoplatelets: Low-Threshold Stimulated Emission Despite
Low Photoluminescence Quantum Yield. ACS Photonics.

[29] Guzelturk B., Erdem O., Olutas M., Kelestemur Y., Demir H. V. (2014). Stacking in Colloidal Nanoplatelets: Tuning Excitonic
Properties. ACS Nano.

[30] Tan M. J. H., Wang Y., Chan Y. (2019). Solution-Based
Green Amplified Spontaneous
Emission from Colloidal Perovskite Nanocrystals Exhibiting High Stability. Appl. Phys. Lett..

[31] Park Y.-S., Roh J., Diroll B. T., Schaller R. D., Klimov V. I. (2021). Colloidal Quantum
Dot Lasers. Nat. Rev. Mater..

[32] Li M., Zhi M., Zhu H., Wu W.-Y., Xu Q.-H., Jhon M. H., Chan Y. (2015). Ultralow-Threshold
Multiphoton-Pumped Lasing from Colloidal Nanoplatelets
in Solution. Nat. Commun..

[33] Philbin J. P., Brumberg A., Diroll B. T., Cho W., Talapin D. V., Schaller R. D., Rabani E. (2020). Area and Thickness
Dependence of
Auger Recombination in Nanoplatelets. J. Chem.
Phys..

[34] Kazes M., Lewis D. y., Ebenstein Y., Mokari T., Banin U. (2002). Lasing from
Semiconductor Quantum Rods in a Cylindrical Microcavity. Adv. Mater..

[35] Wang Y., Leck K. S., Ta V. D., Chen R., Nalla V., Gao Y., He T., Demir H. V., Sun H. (2015). Blue Liquid Lasers
from Solution of CdZnS/ZnS Ternary Alloy Quantum Dots with Quasi-Continuous
Pumping. Adv. Mater..

[36] Maskoun J., Gheshlaghi N., Isik F., Delikanli S., Erdem O., Erdem E. Y., Demir H. V. (2021). Optical Microfluidic
Waveguides and Solution Lasers of Colloidal Semiconductor Quantum
Wells. Adv. Mater..

[37] Moore L. A., Smith C. M. (2022). Fused Silica as
an Optical Material [Invited]. Opt. Mater. Express,
OME.

[38] Lu K., Chen Z., Chen H., Zhou W., Zhang Z., Tsang H. K., Tong Y. (2024). Empowering High-Dimensional Optical
Fiber Communications with Integrated Photonic Processors. Nat. Commun..

[39] Zuo J., Lin X. (2022). High-Power Laser Systems. Laser
& Photonics
Reviews.

[40] Steinke M., Spelthann S., Rühl A., Ristau D. (2021). Absorption and Multi-Phonon
Quenching in Nanocrystal Doped SiO_2_ Fibers. Opt. Mater. Express, OME.

[41] Zhu Z., Sun S., Chai X., Gao J., Lu M., Wu Z., Gao Y., Feng T., Bai X., Zhang Y., Yan F., Yu W. W., Ke C. (2024). Quantum Dot-Doped
Optical Fibers. Laser & Photonics Reviews.

[42] Chemnitz M., Junaid S., Schmidt M. A. (2023). Liquid-Core
Optical Fibers—A
Dynamic Platform for Nonlinear Photonics. Laser
& Photonics Reviews.

[43] Chemnitz M., Gebhardt M., Gaida C., Stutzki F., Kobelke J., Limpert J., Tünnermann A., Schmidt M. A. (2017). Hybrid Soliton Dynamics
in Liquid-Core Fibres. Nat. Commun..

[44] Scheibinger R., Hofmann J., Schaarschmidt K., Chemnitz M., Schmidt M. A. (2023). Temperature-Sensitive
Dual Dispersive Wave Generation of Higher-Order Modes in Liquid-Core
Fibers. Laser & Photonics Reviews.

[45] Zhang H., Jiao K., Hu Y., Huang Z., Wu Y., Song Q., Ta V. D., Sun H., Shen H., Wang Y. (2025). A New Generation of Multimilliwatt-Class
Colloidal Quantum-Dot Lasers
at Full Colors. Adv. Mater..

[46] Li Q., Lian T. (2017). Area- and Thickness-Dependent
Biexciton Auger Recombination in Colloidal
CdSe Nanoplatelets: Breaking the “Universal Volume Scaling
Law.”. Nano Lett..

[47] Bertrand G. H. V., Polovitsyn A., Christodoulou S., Khan A. H., Moreels I. (2016). Shape Control
of Zincblende CdSe Nanoplatelets. Chem. Commun..

[48] Wohlfarth, C. Refractive Index of 1,1,2,2-Tetrachloroethene: Datasheet from Condensed Matter. Optical Constants; Springer: Berlin, 2017; Vol. 50. 10.1007/978-3-662-49236-9_73.

[49] She C., Fedin I., Dolzhnikov D. S., Demortière A., Schaller R. D., Pelton M., Talapin D. V. (2014). Low-Threshold
Stimulated
Emission Using Colloidal Quantum Wells. Nano
Lett..

[50] Macias-Pinilla D. F., Planelles J., Climente J. I. (2022). Biexcitons in CdSe Nanoplatelets:
Geometry, Binding Energy and Radiative Rate. Nanoscale.

[51] Jasrasaria D., Weinberg D., Philbin J. P., Rabani E. (2022). Simulations of Nonradiative
Processes in Semiconductor Nanocrystals. J.
Chem. Phys..

[52] Mutyala C., Khan A. H., Leemans J., Rodà C., Hens Z., Moreels I. (2025). Impact of a CdS Crown
on Optical
Gain in CdSe/CdS Core/Crown Nanoplatelets. Advanced
Optical Materials.

[53] Grim J. Q., Christodoulou S., Di Stasio F., Krahne R., Cingolani R., Manna L., Moreels I. (2014). Continuous-Wave Biexciton Lasing
at Room Temperature Using Solution-Processed Quantum Wells. Nat. Nanotechnol..

[54] Diroll B. T., Guzelturk B., Po H., Dabard C., Fu N., Makke L., Lhuillier E., Ithurria S. (2023). 2D II-VI Semiconductor
Nanoplatelets: From Material Synthesis to Optoelectronic Integration. Chem. Rev..

[55] Geiregat P., Tomar R., Chen K., Singh S., Hodgkiss J. M., Hens Z. (2019). Thermodynamic Equilibrium
between Excitons and Excitonic Molecules
Dictates Optical Gain in Colloidal CdSe Quantum Wells. J. Phys. Chem. Lett..

[56] Kozma I. Z., Krok P., Riedle E. (2005). Direct Measurement
of the Group-Velocity
Mismatch and Derivation of the Refractive-Index Dispersion for a Variety
of Solvents in the Ultraviolet. J. Opt. Soc.
Am. B, JOSAB.

[57] Malitson I. H. (1965). Interspecimen
Comparison of the Refractive Index of Fused Silica. J. Opt. Soc. Am., JOSA.

[58] Geilen A., Popp A., Das D., Junaid S., Poulton C. G., Chemnitz M., Marquardt C., Schmidt M. A., Stiller B. (2023). Extreme Thermodynamics
in Nanolitre Volumes through Stimulated Brillouin-Mandelstam Scattering. Nat. Phys..

