# Supplementary material for: Stimulated Emission from 2D CdSe/CdS Nanoplatelets Integrated in a Liquid-Core Fiber
Source: Nano Lett. 2026 Mar 6;26(18):6019–25. doi: 10.1021/acs.nanolett.5c05747 (PMC13178129; doi:10.1021/acs.nanolett.5c05747)
Supplement: Supplementary file 1 [file nl5c05747_si_001.pdf]

Supporting Information

Stimulated Emission from 2D CdSe/CdS Nanoplatelets Integrated in a Liquid-Core Fiber

Veronika Adolfs[1,2,‡], *Dominik A. Rudolph*[2,3,4,‡], *Simon Spelthann*[1,2,†,*], *Artsiom Antanovich*[3,4], *Dan H. Chau*[1], *Mario Chemnitz*[5,6], *Markus A. Schmidt*[5,7], *Jannika Lauth*[2,3,4,8,*], *Michael Steinke*[1,2,9,*]

[1]Leibniz University Hannover, Institute of Quantum Optics, Welfengarten 1, D-30167 Hannover, Germany

[2]Cluster of Excellence PhoenixD (Photonics, Optics, and Engineering – Innovation Across Disciplines), Welfengarten 1A, D-30167 Hannover, Germany

[3]Leibniz University Hannover, Institute of Physical Chemistry and Electrochemistry, Callinstraße 3A, D-30167 Hannover, Germany

[4]Leibniz University Hannover, Laboratory of Nano and Quantum Engineering (LNQE), Schneiderberg 39, D-30167 Hannover, Germany

[5]Leibniz Institute of Photonic Technology, Albert-Einstein-Straße 9, D-07745 Jena, Germany

[6]Institute of Applied Optics and Biophysics, Philosophenweg 7, D-07743 Jena, Germany

[7]Otto Schott Institute of Material Research, Fraunhoferstraße 6, D-07745 Jena, Germany

[8]University Tübingen, Institute of Physical and Theoretical Chemistry, Auf der Morgenstelle 18, D-72076 Tübingen, Germany

[9]Leibniz University Hannover, QUEST-Leibniz-Research School, Callinstraße 36, D-30167 Hannover, Germany

‡ Authors contributed equally
† Current address: Ruhr-University Bochum, Simply Complex Lab, Universitätsstraße 150, D-44801 Bochum, Germany
* Corresponding authors

Table of contents

S1 – NPL synthesis

S1.1 – Materials

S1.2 – Cadmium myristate precursor

S1.3 – Synthesis of 4.5 ML CdSe core-only NPLs

S1.4 – TOP-S solution

S1.5 – CdS crown growth

S2 – NPL characterization

S2.1 – Transmission electron microscopy and size distribution

S2.2 – Absorption and photoluminescence

S2.3 – Atomic absorption spectroscopy and NPL concentration

S3 – Fiber fabrication and characterization

S3.1 – Fiber fabrication

S3.2 – Scanning electron microscopy

S3.3 – Overview of parameters of used LCFs and solvents

S3.4 – Fiber loss measurements

S4 – Details of the experimental setup

S5 – Additional discussion on the NPL gain in a 3-level and 4-level system

S6 – Additional results and analysis of fiber emission

S6.1 – Modes as possible explanation for the red-shifted emission

S6.2 – Biexciton-to-exciton ratio

S6.3 – Hexane-filled non-waveguiding fiber

S6.4 – TCE-filled fiber with 29 µm core

S6.5 – Fitted slopes

S7 – Additional experiments with different NPL concentrations

S1 – NPL synthesis

S1.1 Materials

Cadmium nitrate tetrahydrate (99.999%, Alfa Aesar), sodium myristate (≥99%, Sigma-Aldrich), methanol (98.8%, Sigma-Aldrich), selenium (99.999%, 200 mesh, Alfa Aesar), 1-octadecene (ODE, 90%, Sigma-Aldrich), oleic acid (90% tech., Sigma-Aldrich), ethanol (97%, Sigma-Aldrich), hexane (≥99%, Sigma-Aldrich), sulfur (99.98%, Sigma-Aldrich), tri-*n*-octylphosphine (TOP, 97%, abcr), cadmium acetate (99.95% anhydrous, Sigma-Aldrich), and tetrachloroethylene (TCE, >99% anhydrous, Sigma-Aldrich).

S1.2 Cadmium myristate precursor

The cadmium myristate precursor was synthesized according to Tessier et al.[1] In separate beakers, 1230 mg of cadmium nitrate was dissolved in 40 mL of methanol and 3130 mg of sodium myristate in 250 mL of methanol. After a few hours of stirring, the solutions were mixed and stirred for at least 10 min. The resulting white solid was filtered and washed with 1-2 L of methanol. Finally, the cadmium myristate was dried under vacuum overnight.

S1.3 Synthesis of 4.5 ML CdSe core NPLs

CdSe NPLs core-only NPLs were synthesized by an adapted protocol from Bertrand et al.[2] First, 590 mg of cadmium myristate and 94 mg of selenium mixed with 120 mL of ODE were degassed for one hour. Afterwards, the solution was heated to 240 °C, while at 195 °C 322 mg of anhydrous cadmium acetate was added. The reaction was stopped after 2.5 min by cooling to room temperature via compressed air flow and the addition of 8 mL of oleic acid at around 150 °C. The dispersion was purified three times with a 2:1 ethanol:hexane mixture, followed by a centrifugation (4000 rcf, 10 min) and subsequent redispersion in hexane.

S1.4 TOP-S solution

A 1 M solution of TOP-S was prepared by dissolving 160.3 mg of sulfur in 5 mL of TOP under a nitrogen atmosphere. To ensure a complete dissolution of sulfur, the mixture was stirred overnight.

S1.5 CdS crown growth

The CdS crown growth followed the approach of Schlosser et al.[3] A hexane dispersion of 4.5 ML CdSe NPLs (n(Cd^{2+}) = 0.19 mmol) was added to 32 mL of ODE together 384 mg cadmium acetate dihydrate and 720 µl oleic acid. After removing hexane and degassing in vacuum (60 °C, 1 h), the mixture was heated to 240 °C. Starting at 215 °C, 800 µL TOP-S solution mixed with 7.2 mL of ODE was injected over 20 min with a syringe pump and kept at 240 °C for an additional 10 min. Then the reaction was cooled quickly to room temperature by

compressed air flow. The purification of the core-crown NPLs follows the same procedure as core-only NPLs. For the solvent exchange, NPLs were precipitated by ethanol with following centrifugation (4000 rcf, 10 min) and then redispersed in anhydrous TCE under an inert atmosphere.

S2 – NPL characterization

S2.1 Transmission electron microscopy and size distribution

TEM images were acquired using a FEI Tecnai G2 microscope with a field emission gun operating at 200 kV. Samples were prepared by drop casting a diluted NPL dispersion onto a carbon-coated copper grid (Quantifoil, 300 mesh). **Figure S1** shows TEM images of core and core-crown NPLs and the corresponding size distributions. The average lateral width and length were calculated from the measurements of >200 NPLs using ImageJ software.

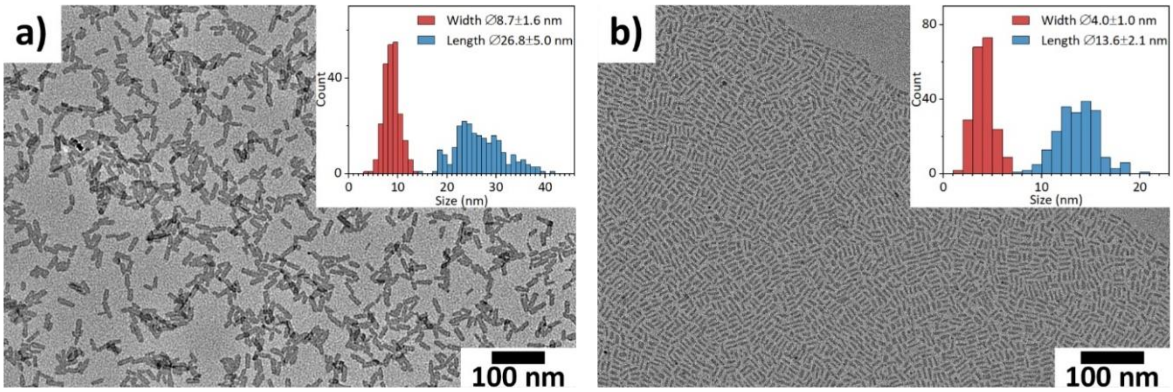

Figure S1. TEM images of CdSe/CdS core-crown NPLs (a) and CdSe core-only NPLs (b). The insets in figures show lateral size distribution histograms of the respective NPLs.

S2.2 Absorption and photoluminescence

For measurements of the absorption and photoluminescence spectra, NPL dispersions were diluted to an optical density of <0.2 at the heavy-hole exciton transition feature in a quartz cuvette (10 mm path length). Absorption spectra were measured with a Cary 5000 spectrophotometer from Agilent Technologies. Photoluminescence spectra, were recorded under 480 nm excitation using Edinburgh FLS1000 photoluminescence spectrometer equipped with an integrating sphere used to determine the quantum yield.

Figure S2 shows photoluminescence spectra of the NPLs dissolved in TCE and hexane. The small shift (<2 meV) between the emission peaks indicates a negligible influence of the solvents' dielectric constant on the screening of the excitons and, consequently, the biexcitons.

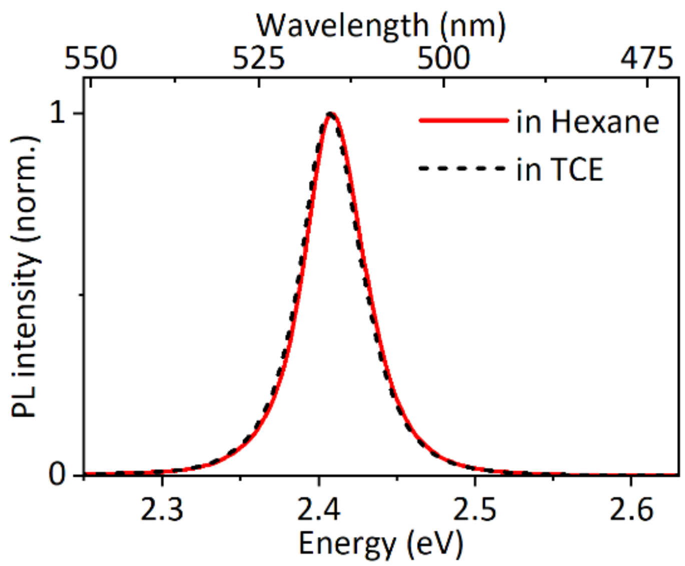

Figure S2. Photoluminescence of the core-crown NPLs dissolved in hexane and in TCE.

Figure S3 shows the absorption spectra of CdSe core-only and CdSe/CdS core-crown NPLs in hexane.

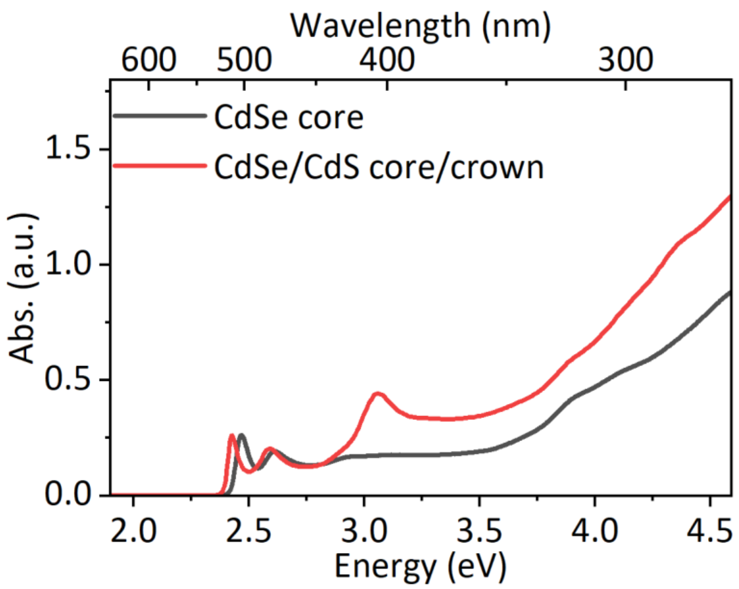

Figure S3. Absorption spectra of CdSe core-only and CdSe/CdS core-crown NPLs in hexane.

For further analysis of the absorption of the core-crown NPLs, we assumed that the absorption A(E) consists of several contributions:

$$A(E) = p_{\mathrm{HH}}(E) + p_{\mathrm{LH}}(E) + C_{\mathrm{HH}}(E) + C_{\mathrm{LH}}(E) + p_{\mathrm{crown}}(E)$$

The first two contributions $p_{\mathrm{HH}}(E)$ and $p_{\mathrm{HH}}(E)$ are related to the absorption of the heavy hole (HH) and light hole (LH) excitons of the CdSe core. Following a commonly used approach,[4] both exciton absorptions were modelled as an asymmetric profile via:

$$p(E) = \frac{A}{2\eta}\left(erf\left(\frac{E - E_X}{\gamma} - \frac{\gamma}{2\eta}\right) + 1\right)\exp\left(\frac{\gamma^2}{(2\eta)^2} - \frac{E - E_X}{\eta}\right)$$

where, E_X is the exciton energy, γ a broadening factor due to thermal effects, and η a localization energy due to allowed violations of the momentum conservation.[5]

The contributions $C_{\text{HH}}(E)$ and $C_{\text{HH}}(E)$ to the absorption are due to the free carrier absorptions and are both modelled as:[4]

$$C(E) = \frac{A_c}{2}\left(\text{erf}\left(\frac{E - E_X - \Delta_x}{\gamma_c}\right) + 1\right)$$

where, Δ_x is the corresponding exciton binding energy and γ_c another thermal broadening parameter.

The last contribution to the absorption ($p_{\text{crown}}(E)$) is due to excitonic absorptions in the CdS crown. We are not interested in its specific features but it certainly overlaps with the free carrier absorption of the LH exciton of the core. Thus, we phenomenologically modelled $p_{\text{crown}}(E)$ as a symmetric Gaussian profile.

The absorption model was fitted to the recorded data and the result is shown in **Figure S4**. The fit was constrained to energies < 3.05 eV, in particular to account for the phenomenologically selection of a Gaussian profile to model the exciton absorptions of the CdS crown.

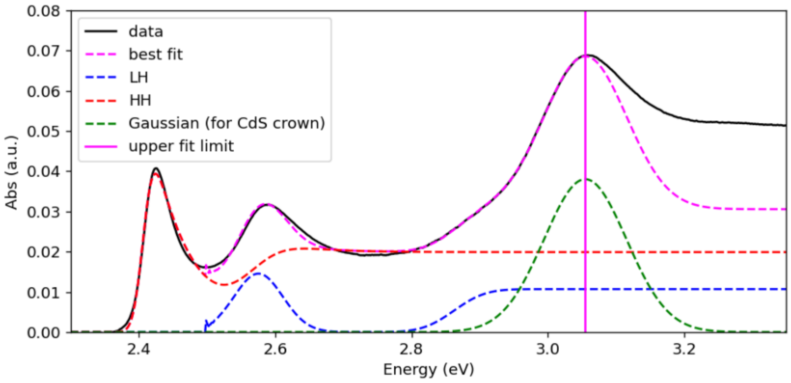

Figure S4. Absorption of the CdSe/CdS core-crown NPLs and the corresponding fit result.

The binding energy of the HH exciton (of the CdSe core) yields the Bohr radius via

$$a_B = \frac{\hbar}{\sqrt{2m_r E_b}}$$

From the fit, the binding energy was determined to be 153 +/- 1.2 meV, which corresponds to a Bohr radius of 1.7 nm.

S2.3 Atomic absorption spectroscopy

To determine the Cd^{2+} concentration of the NPL colloidal solution, a 10 µL aliquot of the solution was dissolved in 1 mL aqua regia overnight. The sample solution was then diluted to 50 ml of Millipore water (R = 18.21 MΩ·cm) in a volumetric flask before measurement. The concentration was determined using a Varian AA140 atomic absorption spectrometer with an air/ethylene (1.5:3.5) flame atomizer and five Cd^{2+} standard solutions with concentrations between 0 and 2.5 ppm for calibration.

S2.4 NPL Concentration

To determine the concentration of NPLs, two approaches were used. For core-only NPLs the relation

$$f_{V,\mathrm{CdSe}} = \frac{\ln(10) \cdot A_{\mathrm{CdSe}}}{\mu_{\mathrm{CdSe}} \cdot L} = \frac{V_{\mathrm{CdSe}}}{V_{\mathrm{solvent}}}$$

is known from literature.[6] Here $f_{V,\mathrm{CdSe}}$ is the volume fraction of CdSe; V_{CdSe} and V_{solvent} are the volume of CdSe and solvent respectively; A_{CdSe} is the measured absorption (at 275 nm); μ_{CdSe} is the intrinsic absorption coefficient of CdSe NPLs and L is the optical path length of the cuvette (1 cm). The total volume of CdSe can be rewritten as $V_{\mathrm{CdSe}} = N_{\mathrm{NPLs}} \cdot V_{\mathrm{core}}$ using the amount of NPLs N_{NPLs} and the volume of the average NPL core $V_{\mathrm{core}} = l_{x,\mathrm{core}} \cdot l_{y,\mathrm{core}} \cdot l_z$. Therefore, the volume fraction equals

$$f_{V,\mathrm{CdSe}} = \frac{N_{\mathrm{NPLs}} \cdot V_{\mathrm{core}}}{V_{\mathrm{solvent}}} = c_{\mathrm{NPLs}} \cdot V_{\mathrm{core}}$$

and the molar NPL concentration can be calculated as

$$c_{\mathrm{NPLs\ (optical)}} = \frac{\ln(10) \cdot A_{\mathrm{CdSe}}}{\mu_{CdSe} \cdot L \cdot V_{\mathrm{core}} \cdot N_{\mathrm{A}}}$$

with Avogadro constant N_{A}. For CdSe/CdS core/crown NPLs, the absorption A_{cc} equals the sum of A_{CdSe} and A_{CdS}, wherein $A_{\mathrm{CdS}} = 0$ at wavelengths $\gtrsim$ 450 nm. Therefore, if optical densities at the heavy-hole exciton transition for core/crown and core-only NPLs are adjusted to be equal, A_{CdSe} at 275 nm can be measured with the core-only NPLs instead of needing to calculate a fraction of A_{cc} (assuming the core size doesn't change during crown growth). In this case c_{NPLs} can be calculated using the above equation. **Table S1** lists the necessary parameters for the calculation. As A_{CdSe} is measured in a cuvette as diluted solution compared to the concentration used in our fiber, the determined concentration must be multiplicated by a factor (34.5 in our case) for the final result. Using this method yields $c_{\mathrm{NPLs\ (optical)}} = 1.56 \pm 0.60$ µmol/L for the

concentration used inside the fibers (corresponding to a semiconductor volume fraction of $f_{V,\mathrm{CdSe/CdS}} = 0.015\%$ for the core/crown NPLs).

As a comparative approach, the concentration of NPLs in the fiber solution

$$c_{\mathrm{NPLs\,(geometrical)}} = \frac{c_{\mathrm{m,NPLs}}}{m_{\mathrm{NPL}}} = \frac{c_{\mathrm{m,Cd^{2+}}} + c_{\mathrm{m,Cd^{2+}}} \cdot \frac{V_{\mathrm{Core}} \cdot M_{\mathrm{Se}}}{V_{\mathrm{cc}} \cdot M_{\mathrm{Cd}}} + c_{\mathrm{m,Cd^{2+}}} \cdot \frac{V_{Crown} \cdot M_{\mathrm{S}}}{V_{\mathrm{cc}} \cdot M_{\mathrm{Cd}}}}{V_{\mathrm{core}} \cdot \rho_{\mathrm{CdSe}} + V_{\mathrm{crown}} \cdot \rho_{\mathrm{CdS}}}$$

was calculated via the mass of an average single NPL m_{NPL} and the mass concentration $c_{m,\mathrm{NPLs}}$ of NPLs in the fiber solution. The latter is based on the mass concentration $c_{m,\mathrm{Cd^{2+}}}$ of Cd^{2+} ions yielded by AAS. Here ρ_{CdSe} and ρ_{CdS} are the densities of bulk CdSe and CdS, while M_{Cd}, M_{Se} and M_{S} are the molar masses of cadmium, selenium and sulfur respectively. The volume of the core of an average NPL $V_{\mathrm{core}} = l_{x,\mathrm{core}} \cdot l_{y,\mathrm{core}} \cdot l_z$ and the volume of an average core-crown NPL $V_{\mathrm{cc}} = l_{x,\mathrm{cc}} \cdot l_{y,\mathrm{cc}} \cdot l_z$ are based on TEM measurements (assuming the core size doesn't change during crown growth). The volume of the average crown $V_{\mathrm{crown}} = V_{\mathrm{cc}} - V_{\mathrm{core}}$ is the difference between the volume of core/crown and their core-only seeds. Using the parameters for this method as listed in **Table S1**, we calculate $c_{\mathrm{NPLs\,(geometrical)}} = 1.12 \pm 0.77$ µmol/L for the concentration used inside the fibers (corresponding to a mass concentration of $c_{\mathrm{m,NPLs}} = 1.02$ g/L).

Both methods yield reasonably comparable results within the measurement error.

Table S1. Parameters used to calculate the NPL concentration.

Parameter	Value	Parameter	Value
A_{CdSe}	0.81	$l_{y,\mathrm{core}}$	4.6 ± 0.8 nm
μ_{CdSe}[7]	$8.87 \cdot 10^5$ cm^{-1}	$l_{x,\mathrm{cc}}$	26.8 ± 5.0 nm
L	1 cm	$l_{x,\mathrm{cc}}$	8.7 ± 1.6 nm
V_{core}	77.3 ± 28.7 nm^3	l_z	1.2 ± 0.1 nm
V_{CC}	279.8 ± 126.9 nm^3	$c_{m,\mathrm{Cd^{2+}}}$	0.73 ± 0.01 g/L
V_{crown}	202.5 ± 155.7 nm^3	ρ_{CdSe}	4.87 g/cm^3
$l_{x,\mathrm{core}}$	14 ± 1.6 nm	ρ_{CdS}	5.66 g/cm^3

S3 – Fiber fabrication and characterization

S3.1 Fiber fabrication

The capillary fibers were drawn in fiber drawing facilities from fused silica tubes at Leibniz Institute of Photonic Technology (IPHT, Jena, Germany) and Hannover Institute of Technology (HITec, Hannover, Germany). The inner capillary diameter of the drawn fiber is determined by different parameters such as the ratio of inner and outer diameter of the initial tube, the heating zone of the furnace of the fiber draw tower under use and the drawing speed. Particularly, the fiber with the inner diameter of 13 µm and the 26 µm were drawn from a HSQ300 grade tubes.

The fiber with the inner diameter of 29 µm was drawn from a F300 grade tube. As outlined in the main text, the initial glass quality impacts the fiber transmission losses, c.f. **section S3.4**.

S3.2 Scanning electron microscopy

SEM images of the capillary fiber with the inner diameter of 26 µm (**Figure 1d** in the main manuscript), were taken by a JEOL JSM-6700F field-emission scanning electron microscope at an accelerating voltage of 2 kV. The fiber sample was cut to a clean edge with a fiber cleaver, thermally coated with carbon to avoid charging effects of the fused silica material, and mounted vertically on a sample holder.

S3.3 Overview of LCF parameters

The numerical aperture

$$NA = \sqrt{n_{\mathrm{Core}}^2 - n_{\mathrm{Cladding}}^2}$$

of any fiber depends on the refractive index of the core n_{core} and the cladding n_{cladding}. For the refractive indices of fused silica and TCE as they are reported in the main text, the resulting NA is 0.38. The V-number, which is a fiber-specific parameter on the number of guided modes, is around 60 for the 26 µm fiber (at 532nm). Since this is significantly larger than the condition for single-mode operation ($V < 2.405$), the 26 µm fiber and, consequently, also the 29 µm fiber operated in a multimode regime.

S3.4 Fiber loss measurements

For fiber loss measurements, one side of the corresponding fiber under test (FUT) was mounted in a 3D-printed custom-made optofluidic mount, which offers an optical window parallel to the fiber facet for convenient free-space laser in-coupling. It also offers microfluidic interfaces, which were connected to standard syringes. For the loss-measurements, the FUT was filled with TCE. A pulsed supercontinuum source (superK, NKT) was coupled into the FUT and the in-coupling was kept stable for the duration of the measurement. The distant end of the FUT was cut-back in subsequent steps (each around 10 cm) several times. After each cut, the FUT transmission was measured by mounting the distant fiber tip inside an integration sphere (diameter 150 mm) coupled to a spectrograph (see **section S4**) via a fiber patch cable. At the wavelength of interest, the fiber loss was computed by fitting Beer-Lamberts law (decaying exponential function) to the data points.

S4 Details of the experimental setup

For the experiments, the hollow core fibers were filled with the NPLs dispersed in the corresponding solvent via a standard syringe and mounted horizontally in the setup. The

elongated 7 cm long pump beam was formed with two cylindrical lenses and focussed on the prepared fibers. To ensure clarity of the signal origin, a short-pass filter with a cutoff at 500 nm was placed in the laser path. A detailed sketch of the setup with all optical elements is shown in **Figure S5**.

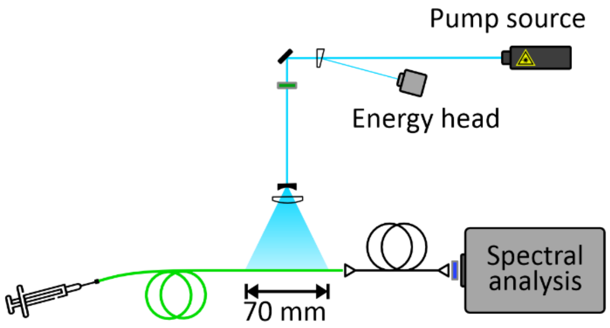

Figure S5. Measurement setup with all optical elements.

The pump laser is a high-power optical parametric oscillator (GWU primoScan ULD/240), based on a Nd:YAG solid-state laser with a repetition rate of 10 Hz and a pulse length of 4 ns. The laser was set to a wavelength of 480 nm for all measurements. The pulse energy is changed by a variable filter and monitored via a pyroelectric energy sensor (Ophir PE9-C), which detects a defined reflex of the incoming pulses provided by a wedge. For the conversion of the monitored energies to the pump (peak) intensity, the power at the fibers position was measured (i.e. calibrated) once with a pinhole with a well-defined diameter of 100 µm. Any emission from the used LCFs was collected directly at the tip of the fiber with a collimator (incl. a VIS anti-reflective coating) and a multimode fiber patch cable. A spectral long-pass 500 nm filter was induced to suppress scattered pump signal. The emission was spectrally analysed by a Shamrock 303i spectrograph (Andor) with a Newton DU970NUV camera (Andor). The settings for these instruments were kept the same for all measurements. The camera was cooled to -65 °C, the spectrographs grating featured 400 L/mm and a blaze wavelength of 400 nm. For an analysis of the spectral resolution obtained for the used setting, the signal of a MHz linewidth 532 nm laser source was fed into the setup. The measured corresponding measured full-width-at-half-maximum (FWHM) (i.e. the resolution) was 17 meV.

S5 – Additional discussion on the NPL gain in a 3-level and 4-level system

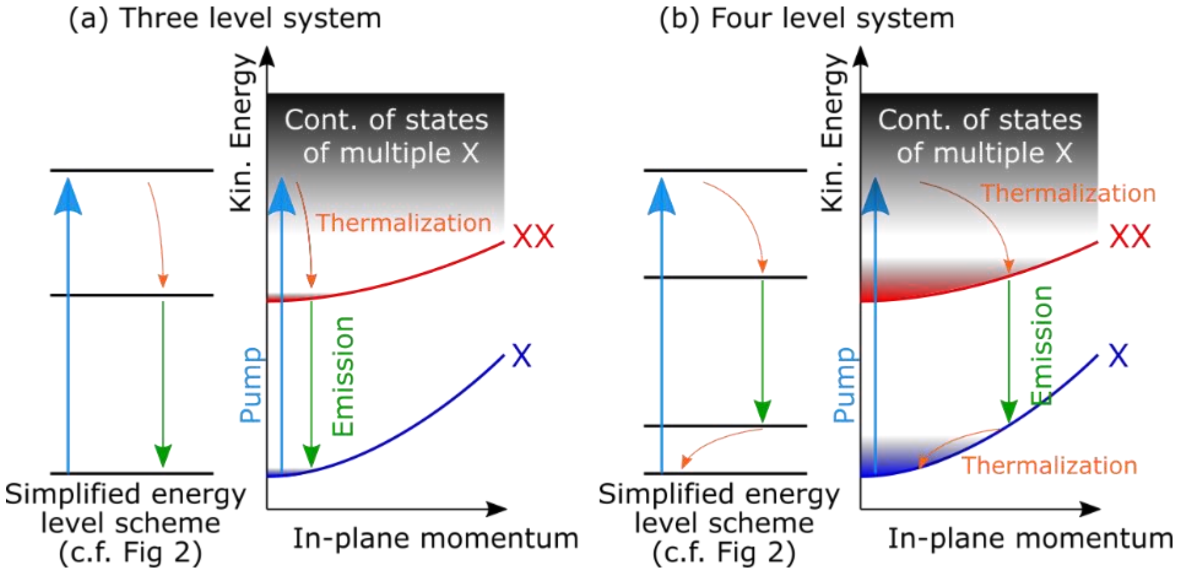

Figure S6. The excitonic and biexcitonic dispersion curves as part of a three-level system (a) and a four-level system (b).

For a 2D NPL, **Figure S6** shows the excitonic and biexcitonic dispersion curves, i.e., the kinetic energy in dependence of the in-plane momentum. Although both dispersion curves are quadratic ($E \propto mk^2$), the proportionality differs, as the (effective) mass of biexciton is twice that of the exciton (neglecting the additional binding energy).

The case of a quite restricted in-plane momentum ($k \approx 0$), either due to a near-zero temperature or an additional spatial (in-plane) confinement as in 0D quantum dots, is shown in **Figure S6a**. If optically pumped above the bandgap, such configuration resembles a conventional 3-level system that can only provide gain if the population (density) of the biexcitonic states exceeds that of the excitonic state (population inversion). As verified by Greiregat et al., since biexcitons and excitons exist in a thermodynamic equilibrium on 2D NPLs, such population inversion requires to pump under extremely high photon rates (pulse energies). This is due to the remaining in-plane degrees of freedom (non-vanishing phase space): pairs of excitons are not forced to immediately form a (bound) biexciton, since there exist plenty of thermodynamically allowed un-bound configurations. This is in stark contrast to quantums dots, in which the 0D confinement enforces two excitons to always form biexcitons.

In comparison, **Figure S6b** shows the case of a non-zero temperature for which the excitonic and biexcitonic kinetic energy states are populated following a Boltzmann distribution. If pumped and considering momentum conservation (for the XX → X transition), such configuration resembles a conventional 4-level system for transitions with sufficiently high in-

plane momentum (see **Figure S6b**). For such 4-level transitions, there are two consequences as outlined in the main manuscript:

1) Since the involved excitonic states are barely populated (via Boltzmann), population inversion and corresponding gain is obtained even at low pumping rates. This contributes to the low threshold we observed even under quasi-CW pumping conditions.
2) The difference in the dispersion slopes yield a red-shift of the emission, as is describedby our experiments and results.

S6 – Additional results and analysis of fiber emission

For convenient comparison, **Figure S7** shows a summary of the recorded spectra from the various experiments. It contains the spectra from the TCE-filled fibers with the inner diameter of 13 µm, 26 µm and 29 µm as well as hexane-filled 26 µm fiber (referred to as "non-waveguiding" in the main text).

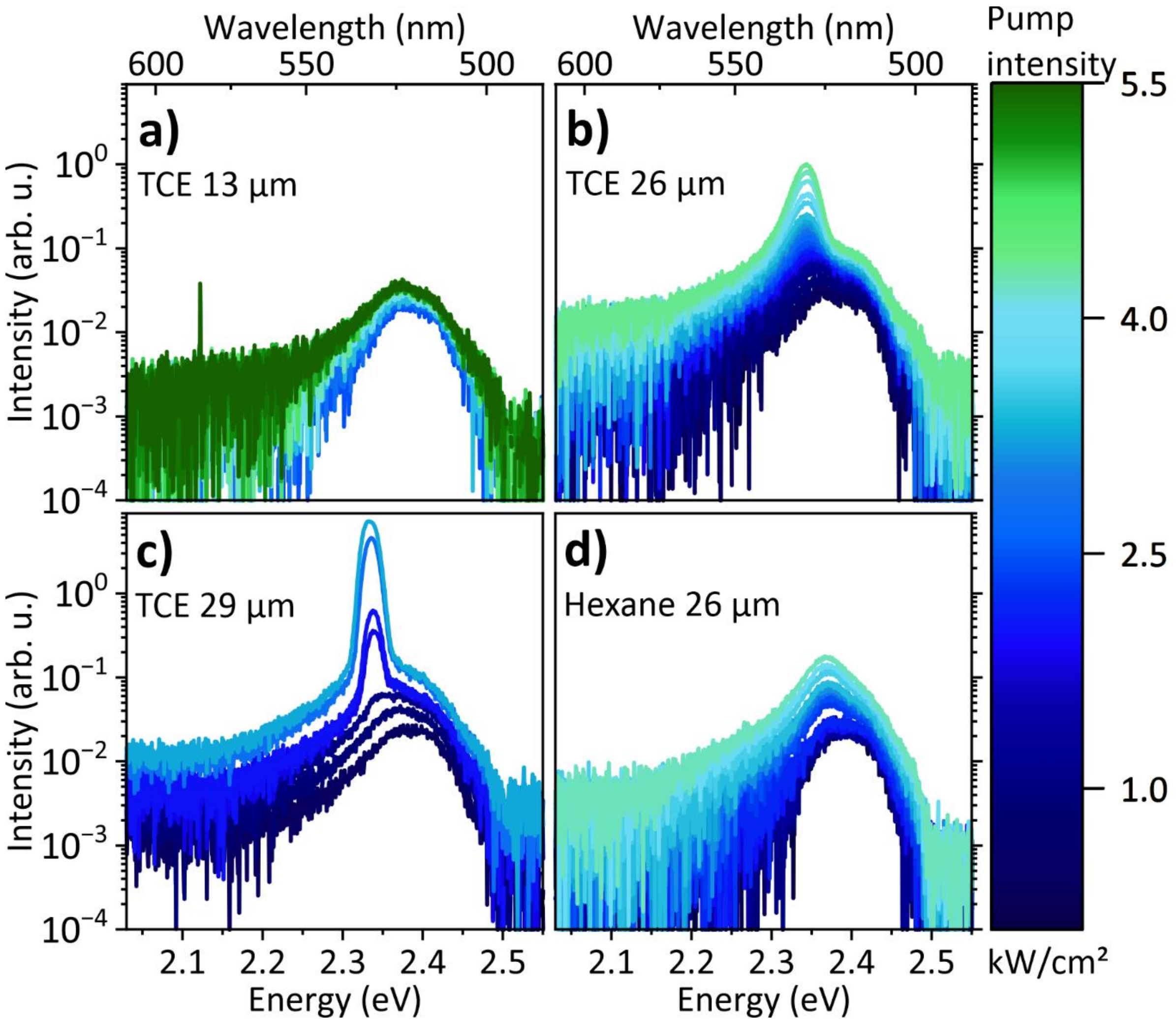

Figure S7. Emission spectra by increasing pump intensities from LCFs filled with CdSe/CdS NPLs in TCE for core diameters of 13 µm (a), 26 µm (b), and 29 µm (c), and from NPLs in hexane in a 26 µm core fiber (d).

S6.1 Modes as possible explanation for the red-shifted emission

As described in the main manuscript, we attribute the red-shift of the biexcitonic component to the ASE onset. In the following, we show that the red-shifted emission cannot be related to any filtering or Purcell effect by the modes in the fiber.

Non-existence of longitudinal modes

There are no distinct (discrete) longitudinal modes in the fiber since there is no cavity around the it. The light propagation through the fiber is broadband (continious) and only limited by the background losses. Thus, it can be ruled out that any spectral filtering (due to a non-continious spectral transmission) is responsible for the observed red-shifted ASE.

Role of transversal modes

As explained in section 3.3, the V number of fibers determines the number (M) of guided transversal modes via the following approximation:

$$M = \frac{4V^2}{\pi^2} + 2$$

For the used solvent, the following table summarizes the corresponding numbers in the wavelength regime 500 – 550 nm (~2.48 eV – ~2.25 eV), which covers the observed emission range.

Table S2. V number and number of guided transversal modes per fiber core diameter.

	13 µm fiber		26 µm fiber		29 µm fiber	
	500 nm	550 nm	500 nm	550 nm	500 nm	550 nm
V	31.0	28.2	62.1	56.4	69.2	62.9
M (rounded)	393	325	1564	1293	1945	1608
Rel. change towards the longer wavelength	-21 %		-21 %		-21 %	

Independent of the fiber core diameter, there is a -21% reduction of the number of transversal modes towards longer wavelengths (lower energies). This means that if some some kind of Purcell effect would appear (by having a higher number of avaliabe photonic states), it would be on the blue (higher energy) side of the spontaneous emission. Thus, such an effect cannot explain the red-shifted emission that we identified as ASE.

S6.2 Biexciton-to-exciton ratio

As outlined in ref. 4, the formation of biexcitons (from excitons) on a NPL follows thermodynamic rules, i.e., biexcitons and excitons co-exist in a thermodynamic equilibrium.

For a given mean number of charge carriers $< N >$ on an NPL, the absolute mean number of excitons $< N_X >$ and biexcitons $< N_{XX} >$ are

$$< N_X > = \frac{\sqrt{8K < N > +1} - 1}{4K}$$

$$< N_{XX} > = \frac{4K < N > + 1 - \sqrt{8K < N > +1}}{8K}$$

The constant K is related to the thermodynamic association/dissociation equilibrium of excitons and biexcitons and is given by

$$K = \frac{4\pi\hbar}{g_X^2 m_X k_B T S} \exp\left(\frac{\Delta_{XX}}{k_B T}\right)$$

where, g_X is the degeneration of the excitonic state, m_X the reduced exciton mass, S the NPL surface area and Δ_{XX} the biexciton binding energy. Using the numbers as summarized in **Table S3** and assuming exemplary temperatures up to 800 K (see next section), the ratio $< N_{XX} >/< N_X >$ in dependency of the mean number of charge carriers $< N >$ is shown in **Figure S8**. As outlined in the main text, for situations with $< N_{XX} >/< N_X >$ less than unity, gain and ASE can only build-up on the red tail of the spontaneous biexciton emission driven by XX-to-X decays with non-zero wave vector.

Table S3. Parameters used to compute the results shown in **Figure S8Figure .**

Parameter	Value	Note
g_X	4	As in ref. 5
m_X	0.086 m_e	Calculated from the effective electron and hole masses reported;[5] m_e is the electron mass.
S	4 x 14 nm	Average size of the CdSe cores, see section 2.1.
Δ_{XX}	30 meV	See main paper.

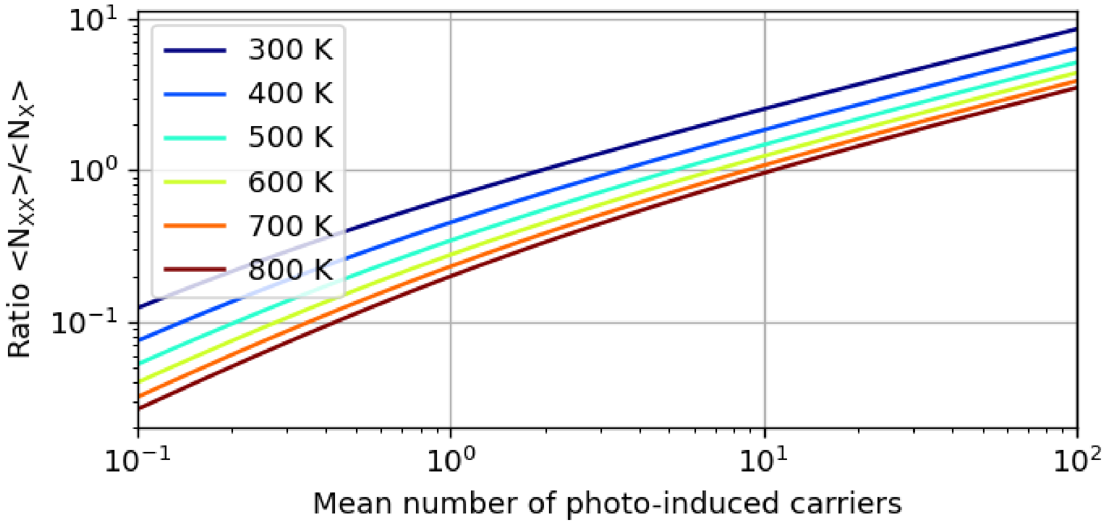

Figure S8. Ratio of biexciton-to-exciton density as a function of the mean number of photoinduced carriers for temperatures from 300 K to 800 K.

For our experiments, predicting the mean number $< N >$ of excited charge carriers would require to numerically solve and average a time-dependent rate equation, taking into account the quasi-CW pump and the intrinsic lifetime of all involved charge carries species. A best-case approximation (maximum feasible $< N >$) can be found, if an infinite lifetime is assumed, i.e., charge carries such as excitons and biexcitons accumulate during the quasi-CW pump pulse without any decays. In that case,

$$< N_{\mathrm{max}} > = \sigma \frac{I_{\mathrm{pump}}}{h\upsilon} \tau_{\mathrm{pump}}$$

with the absorption cross section σ of the NPLs, Planck's constant h, the frequency of the pump υ, the pump (peak) intensity I_{pump}, and the pump pulse length τ_{pump}. Assuming an absorption cross section of around $5 \cdot 10^{-14}$ cm^2,[8] we found that $< N_{\mathrm{max}} >$ is less than 3.5 even at the highest intensities we used. Thus, this finding strongly indicates that our experiments operated in regimes where $< N_{XX} >/< N_X >$ is less than unity.

S6.3 Hexane-filled non-waveguiding fiber

The spectra obtained from the non-waveguiding hexane-filled fiber (26 µm Ø) were fitted with the same two-component model as for the other experiments. The fitted exciton and biexciton center energies are shown in **Figure 3b** of the main text. Besides, **Figure S9** shows the spectrum obtained at a pump intensity of 0.95 kW/cm^2 and the fit result.

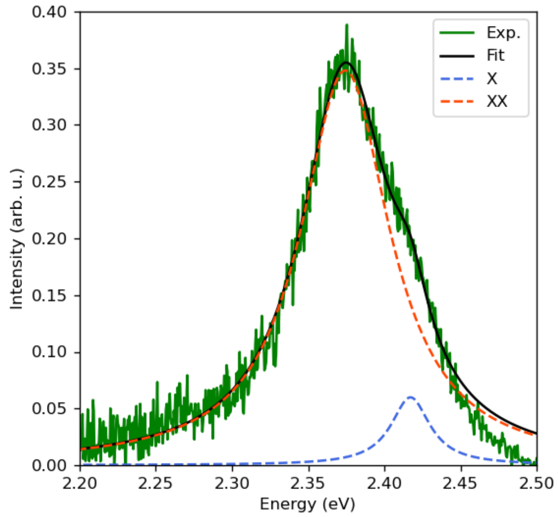

Figure S9. Exemplary data for the X and XX fit of the spectra from the hexane-filled fiber at a pump intensity of 0.95 kW/cm^2.

The low energy tail of the recorded spectra follows an exponential decay, as can been seen in **Figure S10a**. Correspondingly, we fitted the tail of all spectra by an exponential Boltzmann decay to compute the effective temperature of the biexcitons. This approach is adapted from Baghani et al.[9] and Pelton et al.[10] where it was used to obtain effective exciton temperatures. The result of the fitting is shown in **Figure S10b**. At low pump intensities, the effective temperature increases rapidly from room temperature to around 800 to 900 K, where it remains for pump intensities above 1 kW/cm^2. Note that the fitted temperature is not necessarily the mean (steady state) temperature of the NPLs but rather the corresponding effective temperature of the biexciton excitation during the pump pulse.

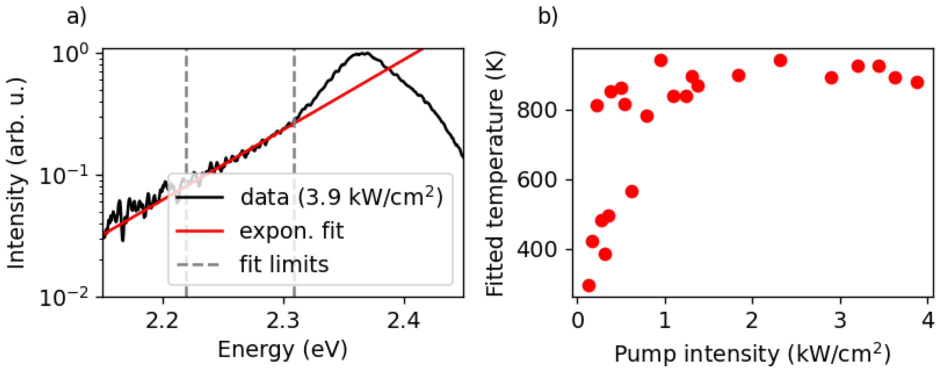

Figure S10. a) Exemplary data for the temperature fits. With the spectrum for 3.9 kW/cm^2 pump intensity (black), the exponential fit (red) and the fit limits (grey dashed lines). b) Fitted effective temperature for the recorded spectra in dependency to the pump intensities.

S6.4 TCE-filled fiber with 29 µm core

As outlined in the main text, the spectra recorded from the 29 µm fiber were fitted with the same two-component model as for the other experiments. **Figure S11** shows exemplary results of this analysis. **Figure S11a** shows the spectrum recorded at a pump intensity of 0.67 kW/cm^2 and the results of the fit. **Figure S11b** shows the slope (integrated spectra) of the fit and the two components. The ASE threshold is at around 0.7 kW/cm^2. **Figure S11c** shows the evolution of the center energies of the exciton and the biexciton components in direct comparison with the corresponding data of the 26 µm fiber. Strikingly, a very similar behavior is observed. Particularly, the X-to-XX separation is around 59 meV at the ASE threshold.

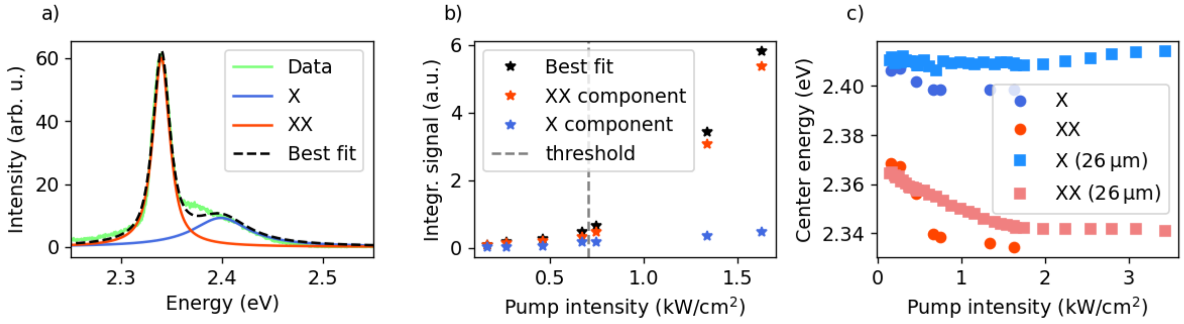

Figure S11. Results of the two-components fits to the spectra recorded from the 29 µm Ø fiber. a) Exemplary spectrum (pump intensity of 0.67 kW/cm^2) and the best fit as well as the two components (X and XX). b) Slopes of the best fit and the two components, indicating an ASE threshold of 0.7 kW/cm^2 (vertical dashed line). c) evolution of the center energies of the exciton and the biexciton components in direct comparison with the corresponding data of the 26 µm diameter fiber.

Figure S12 shows the direct comparison of the FWHM evolution in fibers with the 26 µm and the 29 µm core diameter. Both fibers feature a significant narrowing of the FWHM, i.e., an increase in spectral coherence, around their respective thresholds.

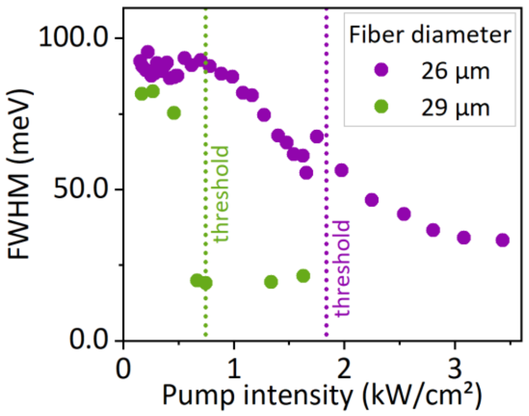

Figure S12. Evolution of the biexcitonic components' FWHM as measured in fibers with a core diameter of 26 µm (purple) and 29 µm (green).

S6.5 Fitted slopes

The linear fits used to obtain the ASE thresholds from the integrated emission intensities of the 26 µm diameter and 29 µm diameter fibers are shown in **Figure S13.** For both fiber core diameters, two sub-datasets were fitted independently. The first sub-dataset for the 26 µm fiber corresponds to datapoints 1 - 25, while the second sub-dataset corresponds to datapoints 26 - 35. The intersection of the corresponding fits is located at 1.8 kW/cm^2. The first sub-dataset for the 29 µm fiber corresponds the first 4 datapoints, while the remaining 3 datapoints correspond to the second sub-dataset. The intersection of the fits is located at 0.7 kW/cm^2.

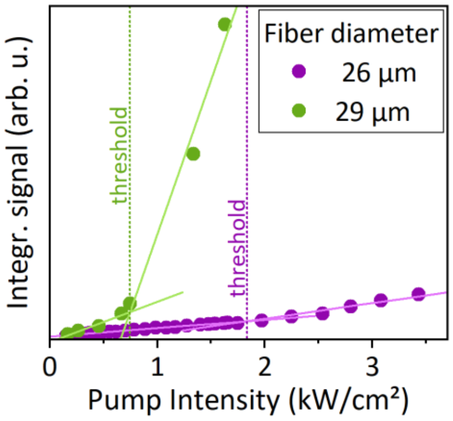

Figure S13. Integrated emission intensity of the fibers with an inner diameter of 26 µm (purple dots) and 29 µm (green dots) and the used linear fits (lines) to obtain the ASE thresholds.

Figure S14 shows the slope obtained with the 26 µm fiber with double logarithmic-scaled axes (and normalized to the y-axis). Below the threshold, the evolution of the slope is sublinear (~$I^{0.6}$). Above the threshold, the scaling is superlinear ($I^{1.6}$). This is in agreement with the CW-pumped system as reported by Grim et al.,[11] who found a similar sublinear scaling below and superlinear scaling above the gain threshold.

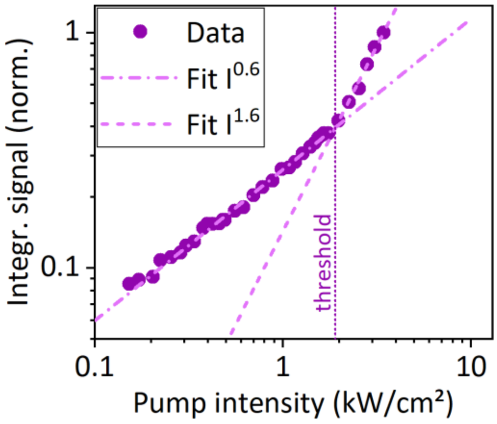

Figure S14. Integrated emission intensity (slope) of the 26 µm fiber (purple dots) shown in a log-log scale.

S7 Additional experiments with different NPL concentrations

As outlined in the manuscript, in addition to the nominal concentration of 1.56 µmol/L (c_1), NPL dispersions with concentrations of 0.78 µmol/L (0.5 × c_1) and 3.12 µmol/L (2 × c_1) were studied in a fiber with a 26 µm core. Normalized emission spectra using all three concentrations are shown in **Figure S15a**, **b,** and **c**. **Figure S15d** shows the slope, i.e., the spectra intergrated from 2.32 eV to 2.36 eV (the spectral window of the biexcitonic emission), of all three concentration measurments. For relative comparison, the x-axis (the pump intensity) is normalized to the ASE threshold obtained with concentration c_1. No ASE threshold can be determined for a concentration 0.78 µmol/L. In contrast, a concentration of 3.12 µmol/L yields an ASE threshold at about 35% with respect to the threshold obtained with concentration c_1.

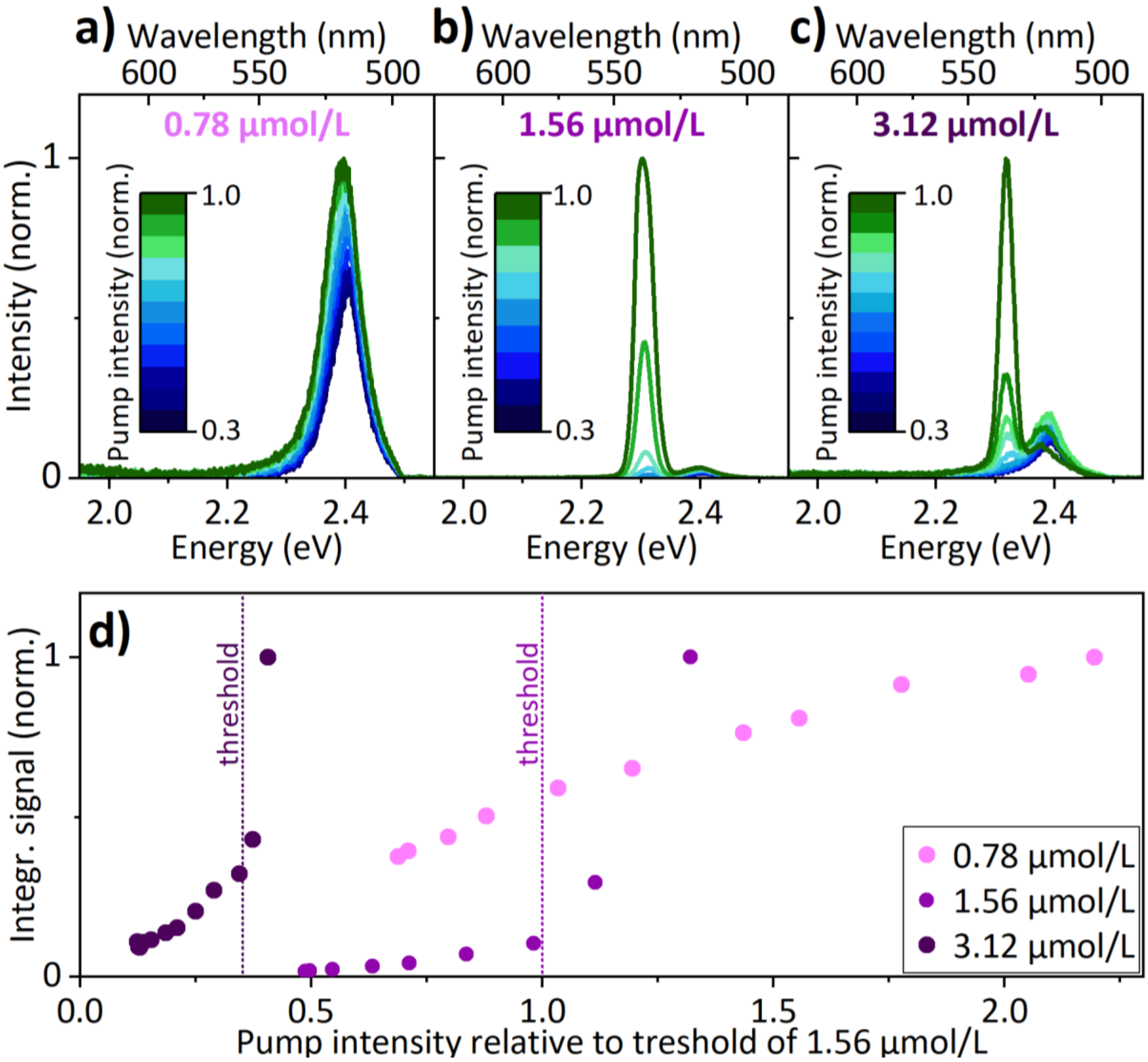

Figure S15. Effects of different NPL concentrations on the ASE threshold in a fiber with an core diameter of 26 µm. Normalized emission spectra using a concentration of 0.78 µmol/L (0.5 × c_1) (a), 1.56 µmol/L (c_1) (b), and 3.12 µmol/L (2 × c_1) (c). d) Normalized slopes of all three concentrations relative to the threshold of the concentration c_1.

References

(1) Tessier, M. D.; Spinicelli, P.; Dupont, D.; Patriarche, G.; Ithurria, S.; Dubertret, B. Efficient Exciton Concentrators Built from Colloidal Core/Crown CdSe/CdS Semiconductor Nanoplatelets. *Nano Lett.* **2014**, *14* (1), 207–213. https://doi.org/10.1021/nl403746p.

(2) Bertrand, G. H. V.; Polovitsyn, A.; Christodoulou, S.; Khan, A. H.; Moreels, I. Shape Control of Zincblende CdSe Nanoplatelets. *Chem. Commun.* **2016**, *52* (80), 11975–11978. https://doi.org/10.1039/C6CC05705E.

(3) Schlosser, A.; Graf, R. T.; Bigall, N. C. CdS Crown Growth on CdSe Nanoplatelets: Core Shape Matters. *Nanoscale Adv.* **2020**, *2* (10), 4604–4614. https://doi.org/10.1039/D0NA00619J.

(4) Geiregat, P.; Tomar, R.; Chen, K.; Singh, S.; Hodgkiss, J. M.; Hens, Z. Thermodynamic Equilibrium between Excitons and Excitonic Molecules Dictates Optical Gain in Colloidal CdSe Quantum Wells. *J. Phys. Chem. Lett.* **2019**, *10* (13), 3637–3644. https://doi.org/10.1021/acs.jpclett.9b01607.

(5) Schnabel, R. F.; Zimmermann, R.; Bimberg, D.; Nickel, H.; Lösch, R.; Schlapp, W. Influence of Exciton Localization on Recombination Line Shapes: $In_xGa_{1-x}As/GaAs$ Quantum Wells as a Model. *Phys. Rev. B* **1992**, *46* (15), 9873–9876. https://doi.org/10.1103/PhysRevB.46.9873.

(6) Achtstein, A. W.; Antanovich, A.; Prudnikau, A.; Scott, R.; Woggon, U.; Artemyev, M. Linear Absorption in CdSe Nanoplates: Thickness and Lateral Size Dependency of the Intrinsic Absorption. *J. Phys. Chem. C* **2015**, *119* (34), 20156–20161. https://doi.org/10.1021/acs.jpcc.5b06208.

(7) Antanovich, A.; Yang, L.; Erwin, S. C.; Martín-García, B.; Hübner, R.; Steinbach, C.; Schwarz, D.; Gaponik, N.; Lesnyak, V. $CdSe_x S_{1-x}$ Alloyed Nanoplatelets with Continuously Tunable Blue-Green Emission. *Chem. Mater.* **2022**, *34* (23), 10361–10372. https://doi.org/10.1021/acs.chemmater.2c01920.

(8) Yeltik, A.; Delikanli, S.; Olutas, M.; Kelestemur, Y.; Guzelturk, B.; Demir, H. V. Experimental Determination of the Absorption Cross-Section and Molar Extinction Coefficient of Colloidal CdSe Nanoplatelets. *J. Phys. Chem. C* **2015**, *119* (47), 26768–26775. https://doi.org/10.1021/acs.jpcc.5b09275.

(9) Baghani, E.; O'Leary, S. K.; Fedin, I.; Talapin, D. V.; Pelton, M. Auger-Limited Carrier Recombination and Relaxation in CdSe Colloidal Quantum Wells. *J. Phys. Chem. Lett.* **2015**, *6* (6), 1032–1036. https://doi.org/10.1021/acs.jpclett.5b00143.

(10) Pelton, M.; Ithurria, S.; Schaller, R. D.; Dolzhnikov, D. S.; Talapin, D. V. Carrier Cooling in Colloidal Quantum Wells. *Nano Lett.* **2012**, *12* (12), 6158–6163. https://doi.org/10.1021/nl302986y.

(11) Grim, J. Q.; Christodoulou, S.; Di Stasio, F.; Krahne, R.; Cingolani, R.; Manna, L.; Moreels, I. Continuous-Wave Biexciton Lasing at Room Temperature Using Solution-Processed Quantum Wells. *Nature Nanotech* **2014**, *9* (11), 891–895. https://doi.org/10.1038/nnano.2014.213.
